# Ammonia Accumulation Drives Intervertebral Disc Degeneration by Triggering Ammonia‐Induced Cell Death Through Lysosome–Mitochondria Crosstalk

**DOI:** 10.1111/cpr.70283

**Published:** 2026-09-27

**Authors:** Yang Zhang, Yucheng Gao, Shuhang Dong, Shuanggong Liu, Lei Liu, Ziyi Song, Jie Xu, Yijun Rong, Guangxu Song, Yingze Zhang, Dongjin Wu, Zengxin Gao

**Affiliations:** ^1^ Department of Spinal Surgery Zhongda Hospital, College of Medicine, Southeast University Nanjing Jiangsu People's Republic of China; ^2^ Department of Orthopedics The Affiliated Hospital of Qingdao University Qingdao Shandong China; ^3^ Department of Orthopaedics The Second Qilu Hospital of Shandong University Jinan Shandong China; ^4^ Department of Orthopedics The Third Affiliated Hospital of Hebei Medical University Shijiazhuang Hebei China

**Keywords:** ammonia metabolism, ammonia‐induced cell death, intervertebral disc degeneration, lysosome–mitochondria crosstalk, organelle homeostasis

## Abstract

Intervertebral disc degeneration (IVDD) is a major cause of low back pain and is closely associated with metabolic imbalance within the disc microenvironment, yet the underlying mechanisms remain poorly understood. Here, we identify ammonia accumulation as a metabolically relevant stressor that promotes nucleus pulposus cell injury and death and characterise ammonia‐induced cell death (AICD) as a non‐canonical, metabolite‐driven pathological process in IVDD. Through a combination of clinical cohort‐based analyses of human samples, multi‐omics profiling, in vitro experiments, and in vivo models, we demonstrate that ammonia stress induces AICD characterised by coordinated disruption of organelle homeostasis. Mechanistically, ammonia accumulation initially impairs lysosomal integrity, which subsequently triggers mitochondrial dysfunction and oxidative stress, accompanied by a metabolic shift from oxidative phosphorylation towards glycolysis, ultimately leading to suppressed mitophagy and activation of cell death pathways. Notably, AICD exhibits crosstalk with multiple programmed cell death pathways, highlighting its integration within the broader cell death network. Importantly, modulation of ammonia levels or restoration of organelle homeostasis effectively alleviates NP cell injury and attenuates IVDD progression. Collectively, these findings support AICD as a previously underappreciated, metabolite‐driven process contributing to IVDD and highlight the ammonia metabolism–organelle axis as a potential target for future metabolism‐ and organelle‐directed therapeutic strategies.

## Graphical abstract

Mechanical strain disrupts the lysosome‐mitochondria axis via GLS1‐mediated ammonia toxicity, causing NP cell death and ECM breakdown in IVDD. CB839‐mediated GLS1 inhibition effectively rescues cellular homeostasis and alleviates disc degeneration.

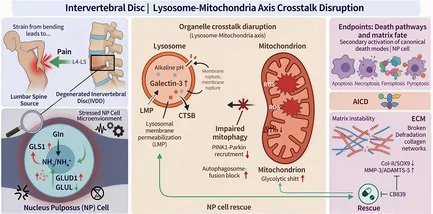

## 1 Introduction

Low back pain (LBP) is one of the leading causes of disability worldwide, affecting approximately 620 million people globally, and is projected to rise to over 840 million cases by 2050 [1, 2, 3]. Intervertebral disc degeneration (IVDD), a principal pathological basis of LBP, has shown an increasing incidence with the ageing population, imposing a substantial burden on patients' quality of life and healthcare systems [4, 5]. Despite advances in surgical and conservative interventions, current treatments remain largely symptomatic and fail to effectively halt or reverse disease progression, largely due to an incomplete understanding of the underlying pathophysiological mechanisms [6, 7]. In recent years, the role of metabolic homeostasis in IVDD has attracted increasing attention, with accumulating evidence indicating that alterations in key metabolic processes, particularly glycolysis and lipid metabolism, critically influence disc cell viability and extracellular matrix homeostasis [8, 9]. However, compared with these well‐characterised metabolic pathways, the contribution of nitrogen metabolism to IVDD remains poorly understood, suggesting a potentially unrecognised role in disc cell stress and degeneration.

Programmed cell death (PCD), including apoptosis, ferroptosis, pyroptosis, necroptosis, and others, plays a fundamental role in maintaining tissue homeostasis and has been widely implicated in diverse pathological conditions [10, 11]. Recently, ammonia‐induced cell death (AICD) has emerged as a distinct, metabolite‐driven cell‐death process initiated by pathological ammonia accumulation and characterised by coordinated lysosomal and mitochondrial dysfunction, thereby expanding the current understanding of metabolic stress‐associated cytotoxicity. AICD was initially characterised in effector CD8^+^ T cells, in which excessive intracellular ammonia accumulates within lysosomes, elevates lysosomal pH, and progressively compromises their capacity for ammonia sequestration. Once lysosomal storage capacity is exceeded, ammonia redistributes to mitochondria, causing mitochondrial swelling and dysfunction, disrupting autophagic flux, and ultimately culminating in cell death. Pharmacological inhibition of glutaminolysis or prevention of lysosomal alkalinisation markedly attenuates this cytotoxicity, highlighting the central role of metabolic–organelle crosstalk in AICD [12]. AICD differs from canonical programmed cell‐death modalities in that it is initiated by ammonia overload and characterised by the consequent disruption of lysosomal–mitochondrial homeostasis. Building upon this mechanistic framework, subsequent investigations have expanded both the regulatory landscape and translational relevance of AICD. It has been demonstrated that extracellular acidosis mitigates ammonia toxicity by modulating its intracellular distribution, thereby conferring a survival advantage to cancer cells under metabolic stress [13]. In parallel, integrative analyses in clear cell renal cell carcinoma have identified AICD‐related gene signatures capable of stratifying patient prognosis and reflecting immune microenvironment characteristics [14]. Consistently, further studies in gastric adenocarcinoma and cancer immunotherapy contexts have linked AICD‐associated metabolic regulators, such as GLS1, to tumour progression, therapeutic responsiveness, and immune modulation [15, 16, 17]. Collectively, these findings position AICD as an emerging nexus connecting metabolic dysregulation to cell fate determination, particularly within tumour and immune contexts. However, despite these advances, the potential involvement of AICD in non‐neoplastic conditions—particularly in degenerative diseases—remains insufficiently understood and largely unexplored.

Ammonia is an inevitable by‐product of amino acid metabolism and becomes cytotoxic when accumulated beyond physiological thresholds [18, 19]. Under normal conditions, systemic ammonia homeostasis is maintained primarily through hepatic detoxification via the urea cycle [20, 21]. In extrahepatic tissues, nitrogen derived from amino acid catabolism is typically incorporated into glutamine and transported to the liver for clearance [22, 23, 24]. However, under metabolic stress or increased biosynthetic demand, certain cells generate ammonia locally through glutaminolysis to sustain tricarboxylic acid cycle anaplerosis [25, 26]. Although auxiliary pathways, including purine and polyamine biosynthesis, may partially consume excess nitrogen, they are insufficient for complete clearance, allowing intracellular ammonia accumulation [27, 28]. The intervertebral disc represents a uniquely susceptible microenvironment in this context. As the largest avascular structure in the human body, it depends on diffusion through the cartilage endplate for nutrient supply and metabolite exchange [29, 30]. Nucleus pulposus cells (NPCs) reside in a chronically hypoxic, nutrient‐limited, and acidic milieu driven largely by glycolysis‐derived lactate [31]. Emerging evidence indicates that metabolic reprogramming in degenerative discs enhances glutamine utilisation; inflammatory stimulation such as IL‐1β promotes glutaminolysis, leading to increased ammonium production and contributing to matrix catabolism [32, 33]. Within this diffusion‐restricted microenvironment characterised by active metabolic reprogramming, glycolysis‐derived lactate predominates in shaping overall acidity, while concurrent ammonia generation may induce localised alkalinising effects, particularly within intracellular compartments such as lysosomes, thereby creating spatial and subcellular pH heterogeneity [34, 35]. With ageing and sustained mechanical loading, endplate calcification further impairs metabolite clearance, establishing a ‘metabolic waste‐retention niche’ that favours the accumulation of cytotoxic metabolites, including ammonia [36, 37]. Given the well‐established capacity of ammonia to disrupt lysosomal integrity and impair mitochondrial function, these features collectively suggest that the intervertebral disc may be particularly susceptible to ammonia‐induced toxicity, thereby providing a mechanistic basis for the potential involvement of AICD in the pathogenesis of IVDD.

In this study, we define the contribution of ammonia to IVDD through an integrated clinical‐to‐mechanistic framework. We show that ammonia accumulation is associated with disc degeneration and is accompanied by profound metabolic reprogramming in NP tissues. Functionally, ammonia stress drives degenerative phenotypes and induces a distinct form of cell death in NPCs. Mechanistically, this process integrates lysosomal dysfunction, mitochondrial impairment, oxidative stress, and defective mitophagy, delineating a lysosome–mitochondria–centred axis underlying ammonia toxicity. Notably, this AICD exhibits features distinct from canonical programmed cell death pathways, highlighting its unique role in NP pathology.

## 2 Materials and Methods

### 2.1 Clinical Cohort and Human NP Tissue Procurement

A prospective observational study was conducted involving 90 consecutive patients with single‐level lumbar disc herniation (LDH) admitted to Zhongda Hospital between November 2024 and June 2025. Patients with spinal tumours, deformities, fractures, or tuberculosis, as well as those with a history of smoking or alcohol consumption, were excluded. Preoperative lumbar magnetic resonance imaging (MRI) was performed in all patients, and the degree of IVDD was independently evaluated by senior spine surgeons with over 25 years of clinical experience using the modified Pfirrmann grading system. Based on this classification, patients were stratified into non‐severe (grade ≤ 3) and severe (grade > 3) degeneration groups. Clinical parameters, including age, sex, body mass index (BMI), serum creatinine (Cr), blood urea nitrogen (BUN), and circulating ammonia levels, were collected for subsequent analyses. Human NP tissues were obtained from patients undergoing spinal surgery for lumbar disc herniation, degenerative disc disease, spinal stenosis, or spondylolisthesis, as well as from adolescent idiopathic scoliosis patients as relatively non‐degenerated controls. NP tissues were categorised according to degeneration severity and used for histological staining and molecular analyses. This study was approved by the Ethics Committee of Southeast University (approval no. 2025ZDKYSB039). Informed consent was obtained from all participants for the collection of clinical data and tissue samples. All procedures involving human specimens were conducted in accordance with the Declaration of Helsinki and relevant ethical guidelines.

### 2.2 Primary Human NPC Isolation and Culture

Human NP tissues were processed immediately after surgical collection under sterile conditions. NP tissues were carefully dissected to remove surrounding annulus fibrosus and ligamentum flavum, minced into ~1–2 mm^3^ fragments, and washed twice with phosphate‐buffered saline (PBS). Tissue digestion was performed in Dulbecco's Modified Eagle Medium (DMEM) containing 2 mg/mL Type II collagenase (Solarbio, Beijing, China) at 37°C for 40 min with continuous agitation. The resulting cell suspension was filtered through a 70‐μm cell strainer, centrifuged at 1000 rpm for 5 min, and resuspended in DMEM/F12 supplemented with 15% foetal bovine serum (FBS) and 1% penicillin–streptomycin. Cells were cultured at 37°C in a humidified incubator with 5% CO_2_.

### 2.3 RNA Isolation and Quantitative Real‐Time PCR (qRT‐PCR)

Total RNA was extracted from NPCs using TRIzol reagent (Thermo Fisher Scientific, Waltham, MA, USA) according to the manufacturer's instructions. RNA concentration and purity were measured using a NanoDrop 2000 spectrophotometer (Thermo Fisher Scientific). Up to 1 μg of RNA was reverse‐transcribed into cDNA using a first‐strand cDNA synthesis kit (Takara, Kyoto, Japan). qRT‐PCR was performed using SYBR Green Master Mix (Takara) on a StepOnePlus Real‐Time PCR system (Applied Biosystems, Foster City, CA, USA). Relative gene expression was normalised to β‐actin and calculated using the 2^−ΔΔCt^ method. The primer sequences are listed in Table S1. Thermal cycling conditions were as follows: 95°C for 30 s, followed by 40 cycles of 95°C for 5 s and 60°C for 30 s. All samples were analysed in triplicate.

### 2.4 Cell Viability Assay

Cell viability was assessed using a Cell Counting Kit‐8 (CCK‐8; Beyotime, Shanghai, China) according to the manufacturer's instructions. NPCs were seeded in 96‐well plates and cultured overnight at 37°C in a humidified incubator with 5% CO_2_. Following the indicated treatments, cells were incubated with DMEM/F12 containing 10% CCK‐8 solution at 37°C for 1 h. Absorbance at 450 nm was measured using a microplate reader (Bio‐Rad, USA). Cell viability was normalised to the control group. All experiments were performed in triplicate.

### 2.5 Lactate Dehydrogenase (LDH) Release Assay

Cytotoxicity was assessed using a LDH release assay. NPCs were seeded in 96‐well plates and subjected to the indicated treatments. Culture supernatants were collected, and LDH activity was measured using a commercial LDH Cytotoxicity Assay Kit (Solarbio, China) according to the manufacturer's instructions. An equal volume of LDH reaction mixture was added to the supernatants and incubated at room temperature for 30 min in the dark. Absorbance at 490 nm was measured using a microplate reader (Bio‐Rad, USA). Cytotoxicity was normalised to the control group. All experiments were performed in triplicate.

### 2.6 Safranin O and Alcian Blue Staining

NPCs were seeded in 24‐well plates and cultured to approximately 80% confluence. Following the indicated treatments, cells were fixed with 4% paraformaldehyde for 15 min at room temperature. Safranin O and Alcian Blue staining were performed using commercial kits (Solarbio, China) according to the manufacturer's instructions. Images were acquired using a Leica light microscope (Leica, Germany).

### 2.7 EdU Incorporation Assay

NPCs were seeded in 6‐well plates and cultured under standard conditions (37°C, 5% CO_2_). Following the indicated treatments, cells were incubated with EdU working solution (Epizyme Biotech, Shanghai, China) for 2 h. Cells were then fixed with 4% paraformaldehyde for 15 min, permeabilised with 0.5% Triton X‐100, and stained using the Click‐iT reaction mixture. Nuclei were counterstained with Hoechst 33342. Images were captured with a Zeiss LSM800 confocal microscope (Germany), and the percentage of EdU‐positive cells was quantified using ImageJ software.

### 2.8 Calcein‐AM/Propidium Iodide (PI) Dual Staining Assay

NPCs were washed twice with PBS and incubated with Calcein‐AM/PI working solution (Beyotime, Shanghai, China) at 37°C in the dark for 20–30 min. Fluorescent images were acquired using a Zeiss LSM800 confocal microscope (Germany) in at least three random fields per sample. The proportion of live (green) and dead (red) cells was quantified using ImageJ software to evaluate cell viability.

### 2.9 TUNEL Assay

NPCs were seeded on coverslips and subjected to the indicated treatments. Cells were fixed with 4% paraformaldehyde for 30 min, permeabilised with 0.1% Triton X‐100, and incubated with TUNEL reaction mixture (Yeasen, Shanghai, China) at 37°C in the dark for 60 min. Nuclei were counterstained with DAPI. Images were captured using a Zeiss LSM800 confocal microscope (Germany) in three random fields per sample, and the apoptotic index was calculated as the percentage of TUNEL‐positive cells.

### 2.10 Flow Cytometry

NPCs were harvested after the indicated treatments, washed twice with PBS, and resuspended in 1× binding buffer. Cells were incubated with Annexin V‐FITC and propidium iodide (PI) in the dark at room temperature for 20–30 min to label apoptotic populations. Following staining, samples were immediately analysed on a BD flow cytometer (BD Biosciences, USA), and data were processed to quantify the percentage of apoptotic cells in each experimental group.

### 2.11 SA‐β‐Gal Staining

Cellular senescence was assessed using a Senescence β‐Galactosidase Staining Kit (Beyotime, Shanghai, China) according to the manufacturer's instructions. NPCs were seeded in 6‐well plates and cultured overnight at 37°C in a humidified incubator with 5% CO_2_. Following the indicated treatments, cells were fixed with the provided fixative solution for 15 min at room temperature. Cells were then incubated with SA‐β‐Gal staining solution overnight at 37°C in a CO_2_‐free environment. Images were acquired using a light microscope, and three random fields per well were analysed. The percentage of SA‐β‐Gal‐positive cells was quantified using ImageJ software.

### 2.12 Immunofluorescent Staining

Paraffin‐embedded rat intervertebral disc sections were deparaffinised, rehydrated, and subjected to antigen retrieval in sodium citrate buffer (0.01 M, pH 6.5). After blocking with 5% bovine serum albumin (BSA) for 1 h at room temperature, sections were incubated with primary antibodies overnight at 4°C, followed by Alexa Fluor‐conjugated secondary antibodies for 1 h at room temperature. Nuclei were counterstained with DAPI‐containing mounting medium. Fluorescence images were captured using a Leica fluorescence microscope (Leica, Germany), and quantitative analysis of fluorescence intensity and positive cells was performed using ImageJ software. For cultured NPCs, cells grown on glass coverslips were fixed with 4% paraformaldehyde for 15 min, permeabilised with 0.5% Triton X‐100 for 20 min, and blocked with 1% bovine serum albumin for 1 h. Cells were then incubated with primary antibodies overnight at 4°C, followed by appropriate fluorescent secondary antibodies for 1 h at room temperature. Nuclei were stained with DAPI for 5 min. Coverslips were mounted and visualised using a Zeiss LSM800 confocal microscope (Zeiss, Germany). Representative images were acquired for subsequent quantitative analysis. Detailed antibody information is provided in Table S2.

### 2.13 Protein Extraction and Western Blot Analysis

Total protein from NPCs and rat intervertebral disc tissues was extracted using radioimmunoprecipitation assay (RIPA) buffer supplemented with protease and phosphatase inhibitors (Thermo, USA). For tissue samples, intervertebral disc tissues were first homogenised on ice prior to lysis. After incubation on ice, lysates were centrifuged at 14,000 × *g* for 10 min at 4°C, and the supernatants were collected as total protein extracts. Protein concentrations were determined using a BCA protein assay kit (Thermo, USA). Equal amounts of protein (30 μg per sample) were separated by SDS–polyacrylamide gel electrophoresis (SDS–PAGE) and transferred onto polyvinylidene difluoride (PVDF) membranes (Bio‐Rad, USA). Membranes were blocked with 5% non‐fat milk in Tris‐buffered saline containing 0.1% Tween‐20 (TBST) for 1 h at room temperature, followed by overnight incubation at 4°C with primary antibodies (see Table S2 for details). After washing with TBST, membranes were incubated with horseradish peroxidase (HRP)‐conjugated secondary antibodies for 1 h at room temperature. Protein bands were visualised using an enhanced chemiluminescence (ECL) detection system and imaged with a Bio‐Rad imaging system. Band intensities were quantified using ImageJ software and normalised to β‐actin.

### 2.14 Untargeted Metabolomics Analysis

Untargeted metabolomics analysis and bioinformatics analysis were performed by Biomarker‐Bio Technology Co. Ltd. (Beijing, China). Human NP tissues were weighed and homogenised using a bead mill. Metabolites were extracted with ice‐cold acetonitrile/methanol/water and subjected to 1 h ultrasonic agitation. After centrifugation at 14,000 × *g* for 15 min, supernatants were collected for analysis. QC samples were prepared by pooling equal aliquots of all extracts. Chromatographic separation was performed on an ACQUITY UPLC HSS T3 column (100 mm × 2.1 mm, 1.8 μm; Waters) using a Vanquish UHPLC system (Thermo Fisher Scientific, Germany), and metabolites were detected on a Q‐Exactive Plus mass spectrometer (Thermo Fisher Scientific). Raw data were processed with Compound Discoverer 3.1 (Thermo Fisher Scientific) for peak detection, alignment, and relative quantification, followed by PCA and OPLS‐DA to evaluate metabolic differences between groups.

### 2.15 RNA Sequencing and Transcriptomic Analysis

RNA sequencing and bioinformatics analysis were performed by LC‐Bio Technology Co. Ltd. (Hangzhou, China). Total RNA was extracted from control and NH_4_Cl‐treated NPCs using TRIzol reagent (Thermo Fisher Scientific, USA). RNA integrity was confirmed (RIN > 7.0) prior to mRNA enrichment, fragmentation, and cDNA library construction. Sequencing was carried out on an Illumina NovaSeq 6000 platform (Illumina, USA) in 2 × 150 bp paired‐end mode. Raw reads were trimmed with Cutadapt to remove adapters and low‐quality bases. Clean reads were aligned to the human reference genome using HISAT2, and transcript abundance was quantified in FPKM via StringTie. Genes with *p* < 0.05 and |fold change| ≥ 2 were considered differentially expressed. DEGs were subjected to Gene Ontology (GO) and KEGG pathway enrichment analyses using DAVID (https://david.ncifcrf.gov/). GSEA, volcano plots, and hierarchical clustering heatmaps were generated using OmicStudio Tools (https://www.omicstudio.cn/).

### 2.16 Proteomics Analysis

Proteomics analysis and bioinformatics analysis were performed by IGENEBOOK‐Bio Technology Co. Ltd. (Wuhan, China). Protein extracts from control and NH_4_Cl‐treated NPCs (*n* = 3 per group) were collected, and protein concentrations were determined using a BCA Protein Assay Kit (Beyotime, China). Proteins were separated by SDS–PAGE, followed by trypsin digestion and desalting. Peptides were labelled using tandem mass tag (TMT) reagents and fractionated by reversed‐phase high‐performance liquid chromatography (HPLC). The resulting fractions were lyophilised and reconstituted for LC–MS/MS analysis. Peptide separation was performed using an EASY‐nLC 1200 system (Thermo Fisher Scientific, USA), and mass spectrometry analysis was conducted on an Orbitrap Exploris 480 (Thermo Fisher Scientific, San Jose, CA) in data‐dependent acquisition (DDA) mode. Raw data were processed using MaxQuant software for protein identification and quantification against the UniProt database, with a false discovery rate (FDR) < 1% at both peptide and protein levels. Differentially expressed proteins were identified for subsequent analyses.

### 2.17 Mito‐Tracker Red and Lyso‐Tracker Green Dual Fluorescent Labeling

NPCs were seeded on glass‐bottom confocal dishes and allowed to adhere. Culture medium was removed, and cells were gently washed with PBS. Cells were incubated with DMEM/F12 medium containing Mito‐Tracker Red (Solarbio, China) and Lyso‐Tracker Green (Solarbio, China) for 20 min at 37°C. Following incubation, cells were washed three times with PBS, and nuclei were counterstained with Hoechst 33342 (Thermo Fisher Scientific, USA) for 20 min, followed by three additional PBS washes. Fluorescence images were acquired using a Zeiss LSM800 confocal laser scanning microscope (Germany). Mitochondrial and lysosomal morphology, as well as colocalisation, were analysed using ImageJ software.

### 2.18 Determination of Lysosomal pH

Lysosomal pH in NPCs was determined using the ratiometric probe Lyso‐Sensor Yellow/Blue DND‐160 (Invitrogen, USA). Following the indicated treatments, cells were incubated with the probe at 37°C in the dark, washed with PBS, and transferred to black 96‐well plates for fluorescence measurement using a microplate reader. The ratio of fluorescence intensity at dual emission wavelengths was calculated and converted to absolute lysosomal pH values based on a standard calibration curve. For calibration, probe‐loaded cells were equilibrated in buffers with defined pH values in the presence of monensin and nigericin to equilibrate lysosomal and extracellular pH. All experiments were performed at least three times independently.

### 2.19 Acridine Orange (AO) Staining

Lysosomal integrity and acidic vesicular compartments in NPCs were evaluated using Acridine Orange (AO) staining (MedChemExpress, USA). Cells were seeded on glass coverslips and treated with NH_4_Cl, with vehicle‐treated cells as controls. After treatment, cells were incubated with AO working solution (1–5 μg/mL) at 37°C in the dark, washed with PBS, and immediately imaged using a fluorescence microscope. Red fluorescent vesicles indicated acidic compartments, and representative images were captured under identical exposure settings. AO‐positive puncta were quantified in randomly selected fields using ImageJ software.

### 2.20 Lysosomal Enzyme Activity Assays

The enzymatic activities of Cathepsin B and β‐hexosaminidase in NPCs were measured using commercial kits according to the manufacturer's instructions. Cathepsin B activity was assessed using a Cathepsin B Activity Assay Kit (Abcam, USA), and β‐hexosaminidase activity was measured using a β‐Hexosaminidase Assay Kit (Sigma‐Aldrich, USA). Briefly, NPCs were seeded in 96‐well plates and treated with the indicated concentrations of NH_4_Cl. After treatment, cells were lysed and incubated with enzyme‐specific substrates following the kit protocols. Absorbance was measured using a microplate reader (Bio‐Rad, USA), and enzymatic activities were calculated according to the kit instructions for subsequent analysis.

### 2.21 JC‐1 Staining for Mitochondrial Membrane Potential

Mitochondrial membrane potential (MMP) in NPCs was assessed using the JC‐1 fluorescent probe (Solarbio, China) following the manufacturer's instructions. Cells were seeded in culture plates and treated as indicated. After treatment, they were washed with PBS and incubated with JC‐1 working solution at 37°C for 20 min. Cells were then washed twice with JC‐1 buffer and imaged using a Zeiss LSM800 confocal microscope (Germany). Red fluorescence indicated healthy mitochondria, whereas green fluorescence indicated depolarised mitochondria. The ratio of green to red fluorescence intensity was quantified in randomly selected fields using ImageJ software.

### 2.22 Intracellular ATP Measurement

Intracellular ATP levels in NPCs were measured using an ATP Bioluminescence Assay Kit (Beyotime, China) following the manufacturer's instructions. After the indicated treatments, cells were lysed in ATP lysis buffer, and lysates were centrifuged at 12,000 × *g* for 5 min at 4°C. Supernatants were incubated with the ATP detection reagent, and luminescence signals were measured using a luminometer. Total protein in each sample was determined with a BCA Protein Assay Kit (Beyotime, China), and ATP levels were normalised to protein content for subsequent analysis.

### 2.23 Seahorse XFe96 Analysis

Mitochondrial respiration and glycolytic activity in NPCs were assessed using a Seahorse XFe96 Extracellular Flux Analyser (Agilent Technologies, CA, USA). NPCs were seeded in XF96 cell culture plates and exposed to ammonia under the indicated conditions. Before measurement, cells were washed and incubated in Seahorse assay medium at 37°C in a non‐CO2 incubator for 1 h. For OCR analysis, oligomycin, FCCP, and rotenone/antimycin A were sequentially injected. For ECAR analysis, glucose, oligomycin, and 2‐deoxy‐D‐glucose were added in sequence. OCR and ECAR were recorded in real time according to the manufacturer's instructions. Data were normalised to cell number or protein content and analysed using Wave software.

### 2.24 Mitochondrial Superoxide Measurement

Mitochondrial superoxide levels in NPCs were assessed using the Mito‐SOX Red mitochondrial superoxide indicator (Yeasen Biotechnology, Shanghai, China) following the manufacturer's instructions. Cells were seeded in confocal dishes and treated as indicated. After treatment, cells were incubated with 5 μM Mito‐SOX at 37°C for 20–30 min, washed twice with PBS, and counterstained with Hoechst 33342. Fluorescence images were captured using a Zeiss LSM800 confocal microscope (Germany), and Mito‐SOX intensity was quantified in randomly selected fields using ImageJ software.

### 2.25 Intracellular ROS Measurement

Intracellular ROS levels in NPCs were assessed using a ROS detection kit (Solarbio, China) following the manufacturer's instructions. Cells were seeded in confocal dishes and treated as indicated. After treatment, cells were incubated with the ROS‐sensitive fluorescent probe at 37°C for 20–30 min, washed twice with PBS, and counterstained with Hoechst 33342. Fluorescence images were captured using a Zeiss LSM800 confocal microscope (Germany), and ROS intensity was quantified in randomly selected fields using ImageJ software.

### 2.26 Autophagic Flux Assessment via mRFP‐GFP‐LC3

Autophagic flux in NPCs was monitored using mRFP‐GFP‐LC3 adenovirus (Genechem, Shanghai, China) following the manufacturer's instructions. Cells were seeded on glass‐bottom dishes and grown to ~50%–60% confluence. Adenoviral particles were added at an optimised MOI and incubated for 2 h, then replaced with fresh medium. After 24–48 h to allow reporter expression, cells were subjected to the indicated treatments, fixed with 4% paraformaldehyde, and imaged using a Zeiss LSM800 confocal microscope (Germany). Autophagosomes (green puncta) and autolysosomes (red‐only puncta) were quantified in randomly selected fields using ImageJ software to evaluate autophagic flux.

### 2.27 Mitophagy Assessment via Mito‐Keima Reporter

Mitophagy in NPCs was monitored using mitochondria‐targeted Mito‐Keima adenovirus (Genechem, Shanghai, China) following the manufacturer's instructions. Cells were seeded on glass‐bottom dishes and transduced at an optimised MOI for 2 h, then replaced with fresh medium. After 24–48 h to allow reporter expression, cells were subjected to the indicated treatments and imaged using a Zeiss LSM800 confocal microscope (Germany). Keima fluorescence in neutral mitochondria and acidic autolysosomes was detected by its distinct excitation spectra, and mitophagic flux was quantified by analysing the red/neutral fluorescence ratio or the number of red puncta in randomly selected fields using ImageJ software.

### 2.28 Field Emission Scanning Electron Microscopy (FE‐SEM) Analysis

NPCs were seeded on sterile 10 mm glass coverslips and fixed with 2.5% glutaraldehyde at room temperature for 1 h. After three PBS washes, cells were post‐fixed in 1% osmium tetroxide for 30 min, dehydrated through graded ethanol, critically point‐dried, and mounted on metallic stubs with carbon adhesive. Samples were sputter‐coated with a thin gold layer and imaged using a FE‐SEM (SU8100, HITACHI, Japan). Representative micrographs were acquired to evaluate cell surface morphology and subcellular structures under different treatments.

### 2.29 Transmission Electron Microscopy (TEM) Analysis

NPCs were collected after treatments and washed twice with cold PBS. Cells were fixed in 2.5% glutaraldehyde at 4°C overnight, followed by post‐fixation in 1% osmium tetroxide in 0.1 M phosphate buffer (pH 7.4) for 2 h at room temperature. Samples were dehydrated through graded ethanol and acetone, infiltrated with acetone/epoxy resin mixtures (1:1 for 2–4 h, 1:2 overnight at 37°C), then embedded in pure epoxy resin and polymerised at 60°C–65°C for ≥ 48 h. Ultrathin sections (70–75 nm) were cut, stained with 2% uranyl acetate and 2.6% lead citrate, and examined using a TEM (HT7800, HITACHI, Japan). Representative micrographs were acquired to assess the integrity of mitochondria, lysosomes, and other subcellular structures in NPCs under different experimental conditions.

### 2.30 Animal Studies and Ethical Approval

All animal procedures were approved by the Animal Experimentation Committee of The Second Qilu Hospital of Shandong University (ethics approval number: KYLL2025694) and conducted in accordance with the Guidelines for the Welfare and Ethical Review of Laboratory Animals (GB/T 35892‐2018, China). Male Sprague–Dawley rats (6 weeks old) were obtained from the Experimental Animal Center and maintained under specific pathogen‐free conditions with a 12 h light/dark cycle and free access to standard chow and water. To establish a chronic systemic ammonia‐overload model, NH_4_Cl was administered using a two‐stage protocol comprising an initial oral‐gavage phase followed by drinking‐water supplementation, adapted from previously reported chronic NH_4_Cl‐loading protocols [38, 39, 40, 41]. After 1 week of acclimatisation, rats were randomly assigned to three groups: control, low‐dose NH_4_Cl, and high‐dose NH_4_Cl. During the initial induction phase (Week 1), NH_4_Cl was dissolved in sterile saline and administered once daily by oral gavage at 100 mg/kg (low‐dose) or 200 mg/kg (high‐dose), while control rats received an equivalent volume of sterile saline via the same route and schedule. All rats had ad libitum access to normal drinking water during this phase. During the subsequent maintenance phase (Weeks 2–9), the low‐ and high‐dose groups were provided drinking water containing 0.28 and 0.56 M NH_4_Cl, respectively, whereas the control group continued to receive normal drinking water. The complete modelling period lasted for 9 weeks. For the rescue experiments, a separate cohort of rats was randomly assigned to five groups: control, ammonia stress model, CB839 alone, NH_4_Cl + CB839, and NH_4_Cl + NAC. Rats in the ammonia stress model, NH_4_Cl + CB839, and NH_4_Cl + NAC groups were subjected to the same high‐dose NH_4_Cl regimen described above. CB839 and NAC administration was initiated after completion of the 1‐week induction phase and continued throughout the subsequent 8‐week maintenance phase. Body weight and general health status were monitored weekly throughout the experimental period. At the end of the 9‐week experimental period, animals underwent in vivo MRI examination of the tails, followed by euthanasia and collection of caudal intervertebral disc tissues for histological and molecular analyses.

### 2.31 MRI Evaluation in Rats

At 9 weeks post‐modelling, MRI scans of the coccygeal intervertebral discs were performed using a 9.4 T small‐animal MRI system (Biospec 94/20 USR, Bruker, Germany) to acquire sagittal T2‐weighted images for evaluating disc signal intensity and structural integrity. IVDD was graded by three independent orthopaedic researchers blinded to the experimental groups, following the Pfirrmann grading system. Relative T2‐weighted grey‐scale intensity was quantified to assess the degree of disc degeneration across different treatment conditions.

### 2.32 Histological Staining of NP Tissues

Human or rat NP tissues were fixed in 4% paraformaldehyde, decalcified in 10% EDTA, dehydrated, paraffin‐embedded, and sectioned at 5 μm. Sections were stained with H&E (Solarbio, China) to evaluate general tissue morphology, Safranin O/Fast Green (Solarbio, China) to assess proteoglycan distribution, Alcian Blue (Solarbio, China) for glycosaminoglycan content, and Masson's trichrome (Solarbio, China) for collagen fibres. Stained sections were imaged using a NanoZoomer S60 slide scanner (Hamamatsu, Japan), and quantitative analysis was performed with ImageJ software. Histological scoring of intervertebral discs was conducted according to the criteria detailed in Table S3.

### 2.33 Immunohistochemistry (IHC) Analysis

Human or rat NP tissues were fixed in 4% paraformaldehyde, decalcified in 10% EDTA, dehydrated, paraffin‐embedded, and sectioned at 4–5 μm. Sections were deparaffinised, rehydrated through graded ethanol, and subjected to antigen retrieval using citrate or sodium citrate buffer at 60°C–98°C. Endogenous peroxidase activity was quenched with 3% hydrogen peroxide, and nonspecific binding was blocked with 5% bovine serum albumin (BSA) for 1 h at room temperature. Sections were incubated with the indicated primary antibodies (see Table S2) at 4°C overnight, followed by incubation with HRP‐conjugated goat anti‐rabbit secondary antibodies for 1 h at room temperature. DAB chromogenic solution was used for visualisation, and nuclei were counterstained with haematoxylin or DAPI for immunofluorescence. High‐resolution images were acquired using a NanoZoomer S60 slide scanner (Hamamatsu, Japan), and quantitative analysis was performed with ImageJ software.

### 2.34 Statistical Analysis

Data are presented as mean ± standard deviation (SD) from at least three independent experiments. Statistical analyses and graphing were performed using GraphPad Prism 9.5 (GraphPad Software, USA). Comparisons between two groups were analysed using an unpaired two‐tailed Student's *t*‐test. For experiments involving three or more groups, one‐way analysis of variance (ANOVA) followed by Tukey's multiple comparisons test was applied. When assumptions of normality or homogeneity of variance were not met, nonparametric tests (Mann–Whitney *U* test for two groups, Kruskal–Wallis test with Dunn's post hoc for multiple groups) were used. Statistical significance was defined as *p* < 0.05.

## 3 Results

### 3.1 Dysregulated Ammonia Metabolism Is Associated With the Progression of IVDD

To determine whether ammonia metabolism is associated with the development of IVDD at the systemic level, circulating ammonia levels were measured in patients with lumbar disc herniation (LDH). Patients were stratified into a severe degeneration group (Pfirrmann Grade IV–V) and a non‐severe degeneration group (Pfirrmann Grade II–III). As summarised in Table 1, serum ammonia levels were significantly elevated in the severe degeneration group, whereas other baseline characteristics, including age, sex, BMI, Cr, and BUN, showed no significant differences between groups. Furthermore, logistic regression analysis identified elevated ammonia levels as an independent risk factor for IVDD (Table 2). These findings indicate a potential link between systemic ammonia metabolism and IVDD progression, prompting further investigation into ammonia metabolic alterations within degenerative disc tissues.

**TABLE 1:** Demographic and clinical characteristics of patients stratified by the severity of IVDD.

Variable	Non‐severe degeneration group (*n* = 45)	Severe degeneration group (*n* = 45)	*t*/*X* ^2^/*Z*	*p*
Age (years)	58.76 ± 7.36	59.24 ± 9.13	−0.280	0.780
Sex				
Male	28	25	0.413	0.52
Female	17	20		
BMI (kg/m^2^)	24.01 ± 3.11	23.31 ± 2.52	1.161	0.249
Cr	82.71 ± 19.81	78.82 ± 17.69	0.982	0.329
BUN	13.24 ± 3.81	12.79 ± 4.01	0.536	0.593
Blood ammonia	27.67 ± 3.19	29.73 ± 4.00	−2.704	**0.008**

*Note:* The bold values indicate statistical significance (*p* < 0.05).

**TABLE 2:** Multivariate logistic regression analysis of factors associated with severe IVDD.

Variables	*β*	S.E.	Wald	df	*p*	OR	95% CI
Blood ammonia	0.181	0.067	7.344	1	**0.007**	**1.198**	**1.051–1.366**
Age	−0.011	0.028	0.166	1	0.684	0.989	0.937–1.044
Sex	0.146	0.452	0.105	1	0.746	1.158	0.477–2.806
BMI	−0.099	0.085	1.360	1	0.244	0.905	0.766–1.070
Cr	−0.013	0.012	1.201	1	0.273	0.987	0.963–1.011
BUN	−0.018	0.059	0.092	1	0.761	0.982	0.875–1.102

*Note:* The bold values indicate statistical significance (*p* < 0.05).

To further determine whether ammonia metabolism is altered locally within degenerative disc tissues, we first evaluated the expression of key ammonia‐metabolising enzymes in human NP samples with different degeneration grades. Representative lumbar MRI images illustrating mild and severe IVDD are shown in Figure 1A. H&E staining revealed pronounced degenerative changes in the severe IVDD group, including disorganised extracellular matrix structure and reduced cellularity compared with mildly degenerated discs (Figure 1B). To evaluate ammonia metabolic activity within degenerative discs, immunohistochemical staining was performed to assess the expression of key ammonia metabolism–related enzymes, including Collagen II, GLS1, and GLUL. Compared with the mild IVDD group, the severe IVDD group exhibited significantly elevated expression of GLS1, whereas Collagen II and GLUL expression was markedly reduced (Figure 1C and Figure S1A). To validate these findings in vivo, we further examined ammonia metabolic alterations in a natural ageing rat model. Histological assessment by H&E and Safranin O–Fast Green staining revealed that caudal intervertebral discs from aged rats exhibited more pronounced degenerative features, including disorganised tissue architecture and reduced proteoglycan content, compared with those from young rats (Figure S1B). Consistent with these structural changes and observations in human tissues, immunofluorescence staining of rat caudal intervertebral discs demonstrated that aged rats exhibited significantly elevated GLUD1 expression and reduced GLUL expression compared with young rats, suggesting that similar ammonia metabolic alterations occur during disc degeneration in vivo (Figure 1D and Figure S1C). To further characterise the metabolic alterations associated with IVDD, we analysed the expression of extracellular matrix metabolism markers together with key ammonia metabolic enzymes in human NP tissues. qPCR analysis revealed that the anabolic markers Col‐II and SOX9 were significantly decreased in severely degenerated discs, whereas the catabolic enzyme MMP‐3 was markedly increased. In parallel, ammonia‐producing enzymes GLUD1 and GLS1 were significantly upregulated, while the ammonia‐detoxifying enzyme GLUL was markedly downregulated (Figure 1E). Western blot analysis further confirmed these changes at the protein level (Figure 1F,G). These findings indicate that enhanced ammonia production and impaired ammonia detoxification accompany extracellular matrix degradation during IVDD progression. To determine whether ammonia stress directly contributes to these metabolic alterations, we established an in vitro ammonia stress model in NPCs using ammonium chloride (NH_4_Cl), which dissociates into NH_4_^+^/NH_3_ and is widely used to mimic ammonia stress in cellular systems. Following treatment with 10 mM NH_4_Cl for 24 h, qPCR and Western blot analyses revealed changes consistent with those observed in degenerative disc tissues: Col‐II and SOX9 were significantly reduced, whereas MMP‐3 was increased. Similarly, ammonia‐producing enzymes GLUD1 and GLS1 were upregulated, while GLUL expression was markedly suppressed (Figure 1H–J). Immunofluorescence staining further confirmed that NH_4_Cl treatment significantly increased GLS1 expression while decreasing GLUL levels in NPCs (Figure 1K and Figure S1D), supporting the notion that ammonia stress can recapitulate the ammonia metabolic imbalance observed in degenerative discs. To characterise metabolic alterations associated with IVDD severity, we performed untargeted metabolomic profiling of human NP tissues obtained from patients with mild or severe disc degeneration and compared the metabolic profiles between the two groups. Principal component analysis (PCA) demonstrated clear clustering of samples within each group, indicating good reproducibility of the metabolomic data (Figure 1L). Similarly, OPLS‐DA analysis further confirmed the separation between groups, and permutation testing supported model stability, with the original model showing higher R^2^ and Q^2^ values than the permuted models, indicating that the model was not overfitted (Figure S2A,B). To further characterise the altered metabolites, we classified them based on their chemical properties. The results showed that these metabolites were distributed across multiple classes, with the most abundant categories including carboxylic acids and derivatives, organooxygen compounds, fatty acyls, and glycerophospholipids. This suggests that metabolic alterations in degenerative NP tissues involve diverse metabolite types (Figure S2C). Volcano plot analysis identified substantial metabolic alterations, with 728 metabolites significantly upregulated and 958 metabolites downregulated in severely degenerated discs compared with mildly degenerated discs (Figure 1M). Consistently, heatmap analysis revealed a distinct metabolic signature between the two groups (Figure 1N). KEGG pathway enrichment analysis further revealed that several metabolic pathways related to nitrogen and amino acid metabolism were significantly altered during IVDD progression, including D‐amino acid metabolism, purine metabolism, pyrimidine metabolism, arginine and proline metabolism, and tryptophan metabolism (Figure 1O). Notably, these metabolic pathways are closely linked to nitrogen turnover and can contribute to increased intracellular ammonia production, suggesting enhanced ammonia metabolic activity within degenerative discs. Collectively, these findings demonstrate that ammonia metabolism is profoundly reprogrammed during IVDD progression, characterised by increased ammonia production and impaired detoxification, and suggest that ammonia accumulation may represent a critical metabolic feature of degenerative discs.

**FIGURE 1:**
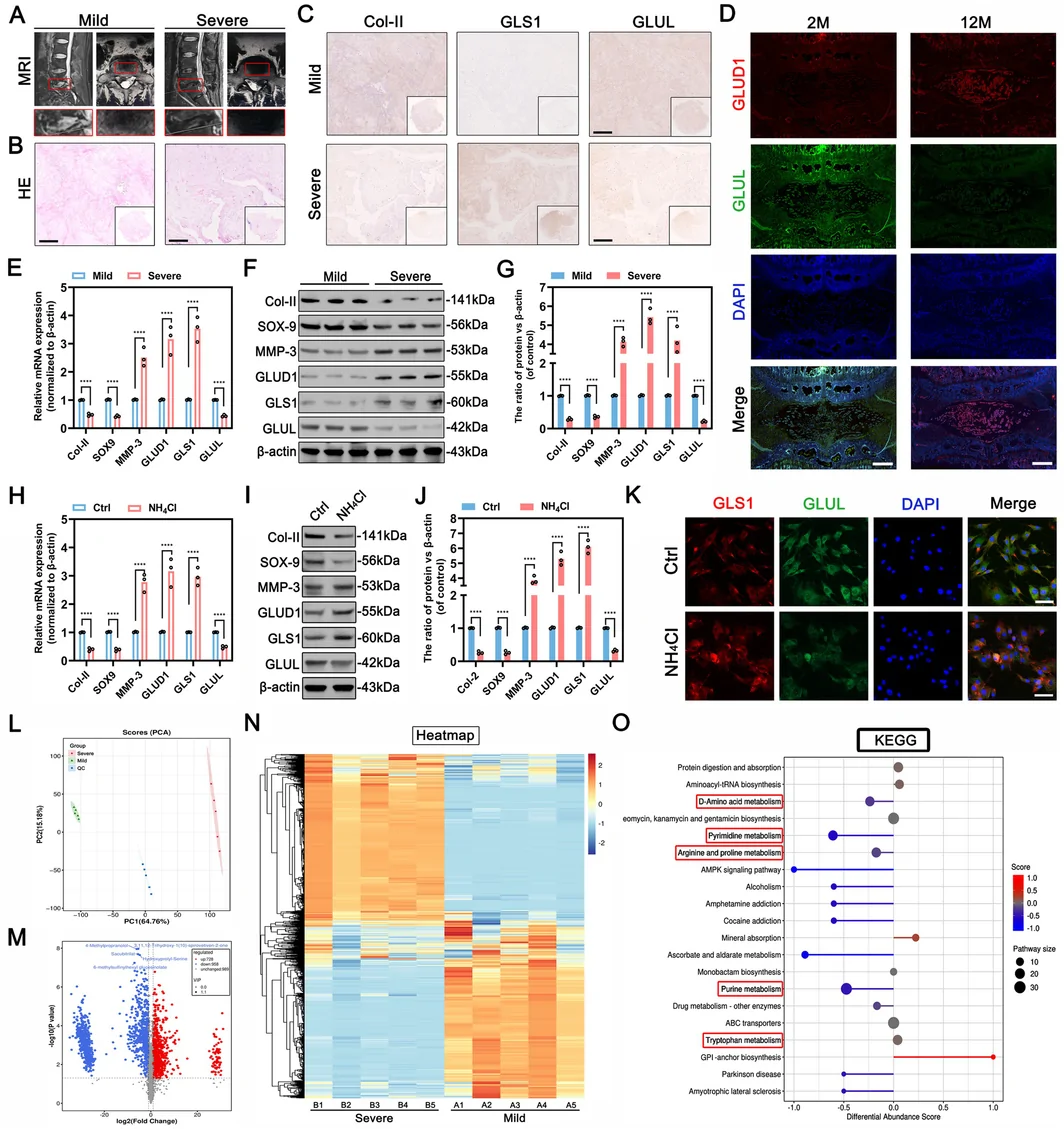
Dysregulated ammonia metabolism and nitrogen‐related metabolic remodelling in degenerative intervertebral discs. (A) Representative lumbar MRI images of patients with mild and severe IVDD based on Pfirrmann grading. (B) H&E staining of NP tissues from mildly and severely degenerated discs. The scale bar represents 100 μm (*n* = 5). (C) Immunohistochemical staining showing the expression of Col‐II, GLS1, and GLUL in human NP tissues (scale bar = 100 μm, *n* = 6). (D) Immunofluorescence staining of GLUD1 and GLUL in caudal intervertebral discs from young (2‐month‐old) and aged (12‐month‐old) rats. Scale bar = 500 μm (*n* = 6). (E) qPCR analysis of extracellular matrix metabolism markers (Col‐II, SOX9, MMP‐3) and ammonia metabolism–related enzymes (GLUD1, GLS1, GLUL) in human NP tissues (*n* = 3). (F, G) Western blot analysis and quantification of extracellular matrix markers (Col‐II, SOX9, MMP‐3) and ammonia metabolism–related enzymes (GLUD1, GLS1, GLUL) in human NP tissues with mild and severe degeneration. (H) qPCR analysis of extracellular matrix markers (Col‐II, SOX9, MMP‐3) and ammonia metabolism–related enzymes (GLUD1, GLS1, GLUL) in NPCs treated with NH_4_Cl. (I, J) Western blot analysis and quantification of extracellular matrix markers (Col‐II, SOX9, MMP‐3) and ammonia metabolism–related enzymes (GLUD1, GLS1, GLUL) in NPCs following NH_4_Cl treatment. (K) Immunofluorescence staining of GLS1 and GLUL in NPCs with or without NH_4_Cl treatment (scale bar = 25 μm). (L) Principal component analysis (PCA) of untargeted metabolomic profiles in NP tissues from mild and severe IVDD groups. (M) Volcano plot showing differentially expressed metabolites between mild and severe IVDD groups. (N) Hierarchical clustering heatmap showing distinct patterns of differentially abundant metabolites between the mild and severe IVDD groups. (O) KEGG pathway enrichment analysis of differentially abundant metabolites. Data are presented as mean ± SD. Statistical significance was determined as indicated. *p < 0.05, **p < 0.01, ***p < 0.001, ****p < 0.0001; ns, not significant.

### 3.2 Ammonia Overload Promotes IVDD In Vivo

To further determine whether ammonia accumulation directly contributes to IVDD progression, an ammonia overload rat model was established to evaluate its effects on intervertebral discs in vivo. A chronic ammonia overload rat model was established through dietary ammonium chloride (NH_4_Cl) administration for 9 weeks, as illustrated in Figure 2A. Rats were randomly divided into three groups: control, low‐ammonia, and high‐ammonia groups to evaluate the effects of ammonia accumulation on caudal IVDD. To ensure that the observed effects were specifically attributable to ammonia overload rather than systemic toxicity, we assessed blood ammonia levels in rats from each group and performed histological evaluation of major organs, confirming effective ammonia elevation without evident multi‐organ damage (Figure S3A,B). MRI of rat caudal intervertebral discs revealed progressive degenerative changes following NH_4_Cl administration (Figure 2B). Compared with the control group, discs from the low‐ and high‐ammonia groups showed progressively decreased T2‐weighted signal intensity, with the most pronounced reduction observed in the high‐ammonia group. Quantitative Pfirrmann grading further confirmed a dose‐dependent increase in degeneration severity (Figure 2C). Histological staining further confirmed progressive structural deterioration of intervertebral discs following ammonia overload. H&E staining showed that control discs exhibited a well‐organised NP and intact annulus fibrosus structure, whereas discs from ammonia‐treated rats displayed progressive structural disruption, including reduced NP area, decreased cellularity, and disorganisation of the annulus fibrosus, which were more pronounced in the high‐ammonia group (Figure 2D). As shown in Figure 2E, Safranin O–Fast Green staining revealed a progressive loss of proteoglycan content in the NP after ammonia exposure, as evidenced by markedly reduced Safranin O staining intensity in the low‐ and high‐ammonia groups. Consistently, Alcian blue staining demonstrated a dose‐dependent reduction in acidic glycosaminoglycan components within degenerative discs (Figure 2F). Representative images in Figure 2G show increased collagen deposition and fibrotic remodelling in ammonia‐treated discs, particularly in the high‐ammonia group. Consistent with these histological findings, quantitative histological scoring further confirmed a significant and dose‐dependent increase in disc degeneration severity in NH_4_Cl‐treated rats compared with controls (Figure 2H). To further evaluate extracellular matrix remodelling, immunohistochemical staining was performed to assess the expression of Col‐II and MMP‐3. Col‐II expression was progressively reduced in discs from NH_4_Cl‐treated rats compared with controls, indicating impaired extracellular matrix synthesis. In contrast, MMP‐3 expression was markedly increased, reflecting enhanced matrix catabolism. Notably, these alterations became more pronounced with increasing NH_4_Cl dosage, with the most evident changes observed in the high‐dose group (Figure 2I,J,M,N). We next examined key enzymes involved in ammonia metabolism. Immunohistochemical staining revealed that GLS1 expression was markedly elevated in NH_4_Cl‐treated discs, whereas the ammonia‐detoxifying enzyme GLUL was significantly reduced compared with controls. Importantly, these changes also exhibited a dose‐dependent pattern, with higher GLS1 expression and further suppression of GLUL observed in the high‐dose NH_4_Cl group (Figure 2K,L,O,P), suggesting a shift towards enhanced ammonia production accompanied by impaired ammonia detoxification. Collectively, these findings demonstrate that chronic ammonia overload accelerates IVDD in vivo, accompanied by extracellular matrix degradation and dysregulation of ammonia metabolism. Together with the observations in human tissues and metabolomic analyses in Figure 1, these results suggest that ammonia accumulation is not merely a metabolic byproduct of IVDD but may actively contribute to the progression of disc degeneration.

**FIGURE 2:**
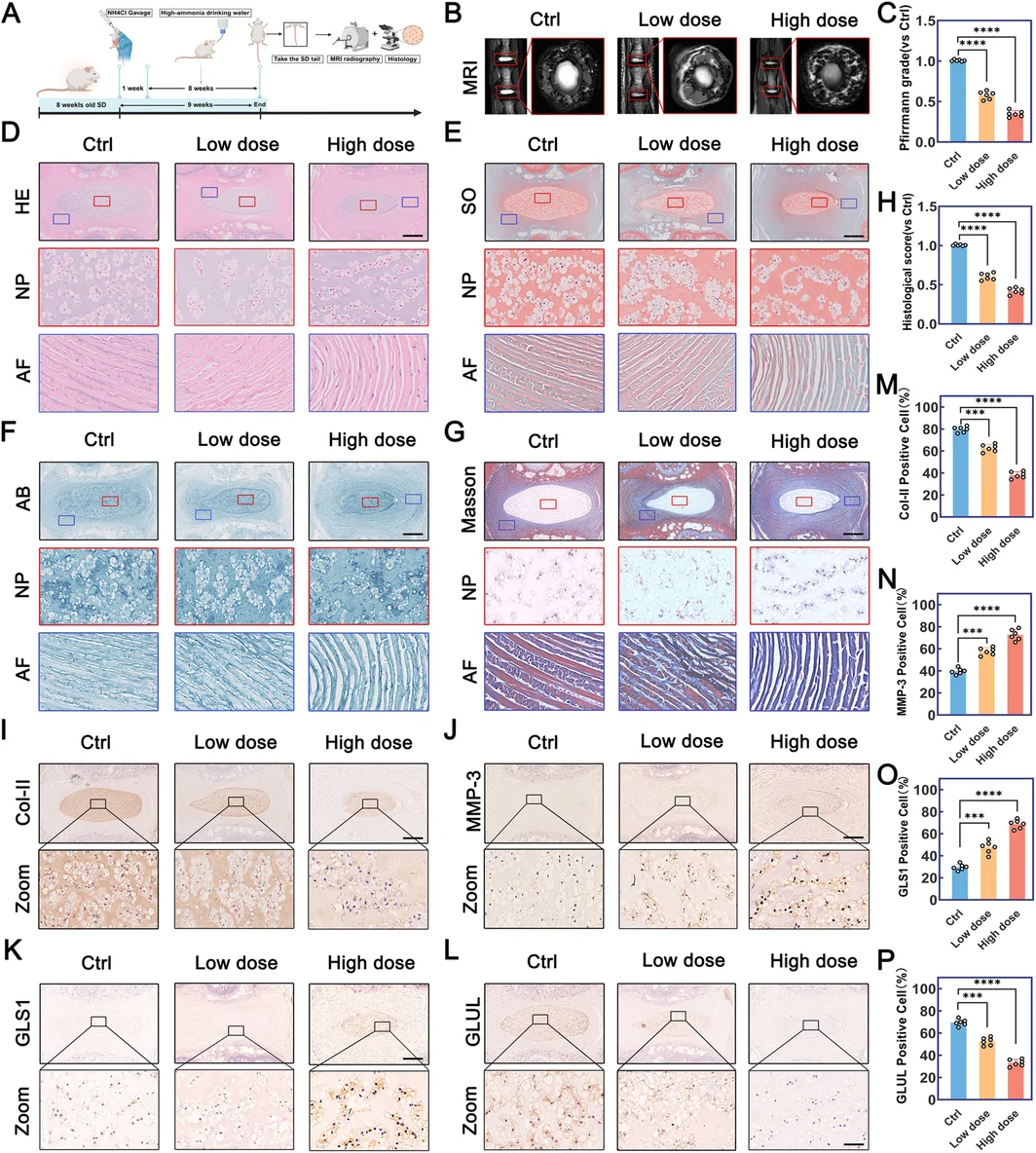
Ammonia overload promotes IVDD in vivo. (A) Experimental schematic of the ammonia overload–induced rat model established by chronic NH_4_Cl administration. (B) Representative T2‐weighted MRI images of rat caudal intervertebral discs in the control and NH_4_Cl‐treated groups. (C) Quantification of T2‐weighted MRI signal intensity and Pfirrmann grading scores of intervertebral discs. (D) Haematoxylin and eosin (H&E) staining showing structural alterations of the NP and annulus fibrosus. (E) Safranin O–Fast Green staining indicating progressive loss of proteoglycan content in the NP. (F) Alcian blue staining demonstrating reduced acidic glycosaminoglycan components in the extracellular matrix. (G) Masson's trichrome staining revealing increased collagen deposition and fibrotic remodelling in degenerative discs. (H) Histological scoring of IVDD. (I, J) Immunohistochemical staining of extracellular matrix–related markers Col‐II and MMP‐3. (K, L) Immunohistochemical staining of ammonia metabolism–related enzymes GLS1 and GLUL in intervertebral disc tissues. (M–P) Quantitative analysis of immunohistochemical staining for Col‐II (M), MMP‐3 (N), GLS1 (O), and GLUL (P). Data are presented as mean ± SD (*n* = 6 per group). Statistical significance was determined by one‐way ANOVA followed by Tukey's multiple comparisons test. *p < 0.05, **p < 0.01, ***p < 0.001, ****p < 0.0001; ns, not significant. Scale bars, 500 μm.

### 3.3 Ammonia Stress Induces Functional Impairment, Apoptosis, and Degenerative Phenotypes in NPCs

To further investigate the direct effects of ammonia accumulation on NPCs, we established an in vitro ammonia stress model using NH_4_Cl, a commonly used method to mimic cellular ammonia overload and hyperammonemic stress in experimental studies. NPCs were treated with increasing concentrations of NH_4_Cl (0, 2, 5, 10, and 20 mM) to evaluate the cellular responses to ammonia stress. We first assessed the impact of ammonia stress on NP cell viability. As shown in Figure 3A, CCK‐8 assays performed at 12, 24, 36, and 48 h revealed a concentration‐ and time‐dependent decline in cell viability following NH_4_Cl treatment. Consistently, LDH release assays demonstrated a dose‐dependent increase in extracellular LDH levels, indicating progressively aggravated cellular injury under increasing ammonia stress (Figure 3B). To further characterise the cellular responses to ammonia exposure, we examined ultrastructural changes using scanning electron microscopy. NH_4_Cl‐treated cells exhibited pronounced morphological alterations, including membrane shrinkage, surface collapse, and the formation of large cytoplasmic vacuoles during cellular contraction (Figure 3C). Notably, this vacuolated morphology is not typically observed in classical forms of cell death, suggesting that ammonia stress may induce a distinct pattern of cellular injury. As shown in Figure 3D, bright‐field microscopy together with Safranin O and Alcian blue staining revealed progressive morphological deterioration and extracellular matrix depletion in NPCs following ammonia exposure, characterised by reduced cell density, structural disruption, and decreased secretion of proteoglycans and acidic glycoproteins. EdU incorporation assays further demonstrated that ammonia stress markedly suppressed NP cell proliferation (Figure 3E and Figure S4A), whereas calcein‐AM/PI staining showed a gradual increase in cell death with rising NH_4_Cl concentrations (Figure 3F and Figure S4B). Collectively, these findings indicate that ammonia stress dose‐dependently impairs NP cell viability, suppresses proliferative capacity, and disrupts extracellular matrix production. Given the pronounced cytotoxicity induced by ammonia, we next investigated whether ammonia stress triggers apoptosis in NPCs. As shown in Figure 3G,H, Western blot analysis revealed a dose‐dependent activation of apoptotic signalling following NH_4_Cl exposure, characterised by progressive upregulation of Bax, cleaved‐caspase‐3, and cleaved‐caspase‐7, accompanied by a marked reduction in the anti‐apoptotic protein Bcl‐2. Consistently, TUNEL staining showed a substantial increase in apoptotic NPCs after ammonia treatment (Figure 3I and Figure S5A). Quantitative flow cytometry analysis further confirmed that apoptotic rates increased progressively with rising NH_4_Cl concentrations (Figure 3J and Figure S5B), while immunofluorescence staining demonstrated enhanced Bax and cleaved‐caspase‐3 expression in ammonia‐treated cells (Figure 3K and Figure S5C). Together, these results indicate that ammonia accumulation robustly induces apoptotic cell death in NPCs. We next assessed whether ammonia stress provokes degenerative and senescence‐associated alterations. As illustrated in Figure 3L,M and Figure S6A,B, NH_4_Cl exposure dose‐dependently suppressed the anabolic markers Col‐II and SOX‐9, while promoting the upregulation of the matrix‐degrading enzymes MMP‐3, MMP‐13, ADAMTS‐4, and ADAMTS‐5, thereby indicating disruption of extracellular matrix homeostasis. In parallel, SASP‐associated cytokines IL‐1β and IL‐6 were markedly elevated (Figure 3N and Figure S7A), and senescence‐associated β‐galactosidase staining demonstrated a pronounced accumulation of senescent NPCs following ammonia exposure (Figure 3O and Figure S7B). Consistently, immunofluorescence staining revealed a progressive decrease in Aggrecan together with marked induction of the catabolic enzyme ADAMTS‐5, confirming the degenerative phenotype (Figure 3P and Figure S7C). To validate these observations in vivo, we examined apoptosis and degeneration markers in the rat ammonia‐overload model. Immunohistochemical analysis revealed markedly increased cleaved‐caspase‐3 and MMP‐13 expression in intervertebral discs from ammonia‐treated rats, with more pronounced changes in the high‐dose group (Figure 3Q,R). Collectively, these findings demonstrate that ammonia stress profoundly disrupts NP cell homeostasis, leading to impaired cell viability, enhanced apoptotic cell death, and the development of degenerative and senescence‐associated phenotypes, suggesting that ammonia accumulation may directly contribute to cellular dysfunction during IVDD progression.

**FIGURE 3:**
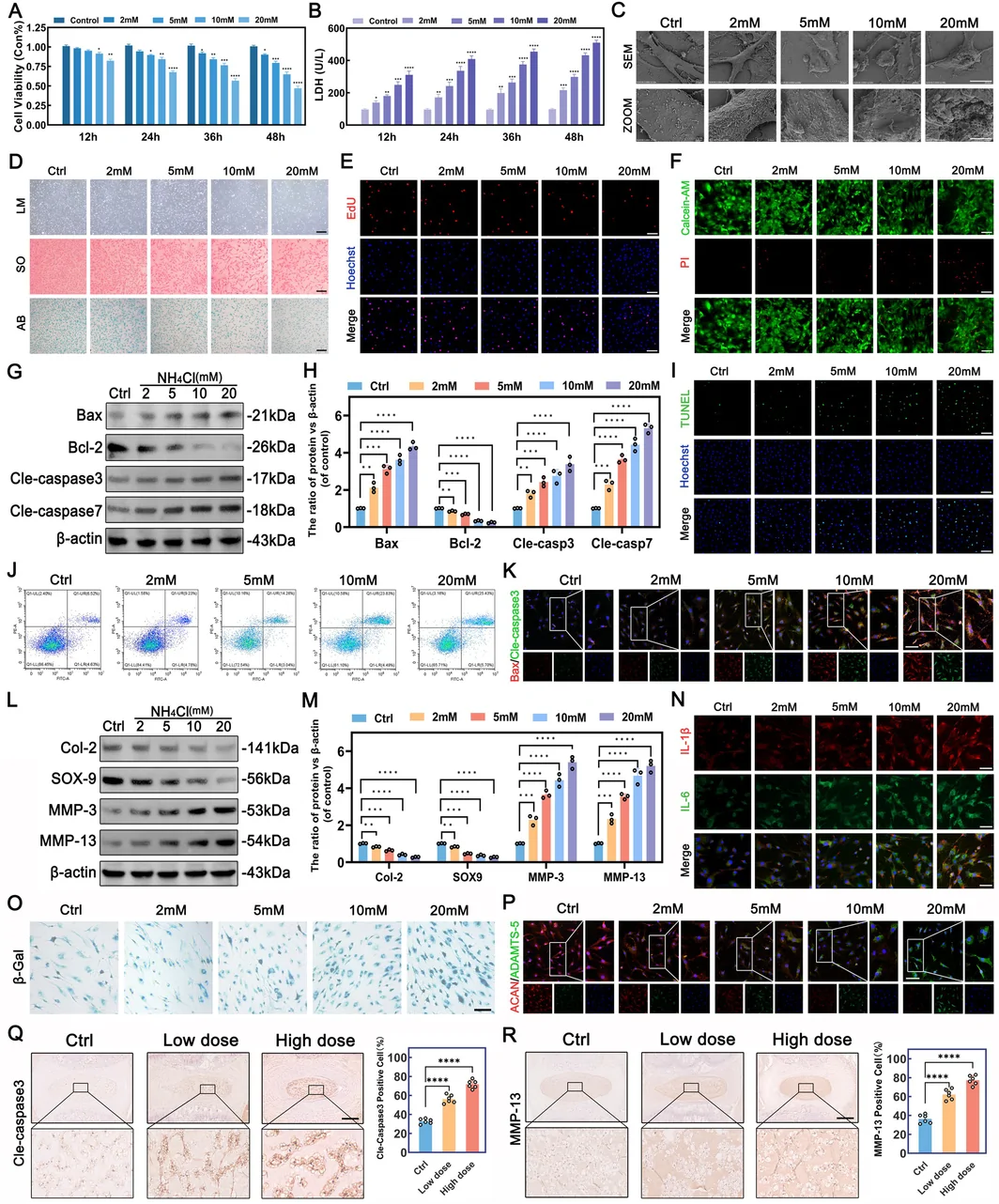
Ammonia stress induces cytotoxicity, apoptosis, and degenerative phenotypes in NPCs. (A) Cell viability of NPCs treated with increasing concentrations of NH_4_Cl for the indicated time points, measured by CCK‐8 assay (*n* = 5). (B) Lactate dehydrogenase (LDH) release assay showing increased cytotoxicity in NPCs following NH_4_Cl exposure (*n* = 5). (C) Representative SEM images showing ultrastructural alterations in NPCs following ammonia exposure, including membrane shrinkage and cytoplasmic vacuolisation. Scale bar, 20 μm; inset magnification, 5 μm (*n* = 3). (D) Bright‐field microscopy images of NPCs stained with Safranin O and Alcian blue showing reduced proteoglycan and acidic glycoprotein production after NH_4_Cl treatment. Scale bar, 100 μm (*n* = 3). (E) EdU incorporation assay showing reduced proliferation of NPCs following ammonia exposure. Scale bar, 50 μm (*n* = 3). (F) Calcein‐AM/propidium iodide (PI) staining showing increased cell death in NPCs under ammonia stress. Scale bar, 50 μm (*n* = 3). (G, H) Western blot analysis and quantitative densitometric analysis of apoptosis‐related proteins (Bax, cleaved‐caspase‐3, cleaved‐caspase‐7, and Bcl‐2) in NPCs treated with NH_4_Cl (*n* = 3). (I) TUNEL staining showing increased apoptotic NPCs following ammonia exposure. Scale bar, 50 μm (*n* = 3). (J) Flow cytometry analysis quantifying apoptosis rates in NPCs treated with increasing concentrations of NH_4_Cl (*n* = 3). (K) Immunofluorescence staining showing increased Bax and cleaved‐caspase‐3 expression in NPCs following ammonia exposure. Scale bar, 25 μm (*n* = 3). (L, M) Western blot analysis and quantification of extracellular matrix anabolic markers (Col‐II and SOX‐9) and catabolic enzymes (MMP‐3 and MMP‐13) in NPCs treated with NH_4_Cl (*n* = 3). (N) Immunofluorescence staining showing increased expression of senescence‐associated secretory phenotype (SASP) factors IL‐1β and IL‐6 in ammonia‐treated NPCs. Scale bar, 25 μm (*n* = 3). (O) Senescence‐associated β‐galactosidase staining showing accumulation of senescent NPCs after ammonia exposure. Scale bar, 50 μm (*n* = 3). (P) Immunofluorescence staining showing decreased Aggrecan and increased ADAMTS‐5 expression in NPCs under ammonia stress. Scale bar, 25 μm (*n* = 3). (Q, R) Immunohistochemical staining showing increased cleaved‐caspase‐3 and MMP‐13 expression in intervertebral disc tissues from rats subjected to ammonia overload (scale bars: 500 μm; *n* = 6). Data are presented as mean ± SD. Statistical significance was determined by one‐way ANOVA followed by Tukey's multiple comparisons test. *p < 0.05, **p < 0.01, ***p < 0.001, ****p < 0.0001; ns, not significant.

### 3.4 Multi‐Omics Profiling Reveals Lysosomal and Mitochondrial Dysfunction and Impaired Autophagy in Ammonia‐Stressed NPCs

To further elucidate the molecular mechanisms underlying ammonia‐induced cellular alterations in human primary NPCs, we performed integrated transcriptomic and proteomic analyses comparing control and NH_4_Cl‐treated cells. The overall multi‐omics analysis workflow is illustrated in Figure 4A. Transcriptomic profiling revealed widespread gene expression changes in NPCs exposed to ammonia stress. Volcano plot analysis identified 2818 upregulated and 2088 downregulated genes in the NH_4_Cl group compared with controls (Figure 4B). Hierarchical clustering further demonstrated a clear separation between groups, indicating substantial transcriptional reprogramming following ammonia exposure (Figure 4C). In the cellular component (CC) category, several enriched terms were associated with mitochondria and lysosomes, including mitochondrion, mitochondrial inner membrane, mitochondrial matrix, mitochondrial intermembrane space, lysosomal membrane, and collagen‐containing extracellular matrix (Figure 4E), suggesting that organelle dysfunction may contribute to ammonia‐induced cellular injury. In the molecular function (MF) category, enrichment analysis highlighted ATP binding, extracellular matrix structural constituent, oxidoreductase activity, and ATP hydrolysis activity (Figure 4F), implying alterations in cellular energy metabolism and redox regulation. Consistently, KEGG pathway analysis identified multiple significantly altered pathways in NH_4_Cl‐treated NPCs, further supporting broad metabolic and stress‐related changes (Figure 4G). Based on the enrichment analyses and previous mechanistic insights, we further examined gene expression signatures associated with lysosomal function, mitochondrial homeostasis, autophagy, and oxidative stress. Heatmap visualisation revealed coordinated alterations in lysosome‐related genes, mitochondrial functional genes, autophagy‐associated genes, and oxidative stress‐related genes, indicating that ammonia stress is associated with disrupted lysosomal and mitochondrial homeostasis, impaired autophagic processes, and altered redox balance (Figure 4H–K). Gene set enrichment analysis (GSEA) provided further support for these findings. Pathways related to late endosome to lysosome transport, autophagosome maturation, mitochondrial electron transport, mitochondrial ATP synthesis coupled proton transport, and energy derivation by oxidation of organic compounds were significantly downregulated. In contrast, pathways associated with response to reactive oxygen species and pro‐inflammatory cytokine production, including positive regulation of interleukin‐1β and interleukin‐6 production, were markedly upregulated (Figure 4L). To validate the transcriptional findings at the protein level, we subsequently performed quantitative proteomic analysis using the same experimental conditions. Volcano plot analysis revealed 190 upregulated proteins and 405 downregulated proteins in NH_4_Cl‐treated NPCs compared with controls (Figure 4M). Hierarchical clustering similarly demonstrated distinct proteomic profiles between the two groups (Figure 4N). GO enrichment analysis of differentially expressed proteins further highlighted biological processes related to oxidative stress response, lysosomal lumen acidification, cellular redox homeostasis, lysosomal protein catabolic process, mitochondrial ATP synthesis, and glutamate metabolism (Figure 4O). Consistent with the transcriptomic data, enrichment in the cellular component category identified significant associations with mitochondria, lysosomes, mitochondrial matrix, and vacuolar proton‐transporting V‐type ATPase complexes (Figure 4P), suggesting coordinated organelle dysfunction. In the molecular function category, altered proteins were enriched in proton‐transporting ATPase activity, oxidoreductase activity, glutathione peroxidase activity, and ATP synthase activity, indicating substantial disruption of cellular redox and energy metabolism (Figure 4Q). KEGG pathway analysis further revealed significant alterations in metabolic and organelle‐associated pathways, including oxidative phosphorylation, lysosome, glutathione metabolism, and central carbon metabolism pathways such as glycolysis/gluconeogenesis and the TCA cycle (Figure 4R). Consistently, GSEA of proteomic datasets demonstrated downregulation of pathways related to mitochondrial matrix function, ATP metabolic processes, mitochondrial protein‐containing complexes, and oxidative phosphorylation, whereas pathways associated with inflammatory response, oxidative stress response, and regulation of cell death were significantly upregulated (Figure 4S). Collectively, integrated transcriptomic and proteomic analyses indicate that ammonia stress disrupts lysosomal and mitochondrial homeostasis, suppresses autophagic processes, and promotes oxidative stress and inflammatory signalling in NPCs. These findings further suggest impaired coordination between mitochondrial and lysosomal pathways, highlighting organelle crosstalk dysfunction as a potential mechanism underlying ammonia‐induced IVDD.

**FIGURE 4:**
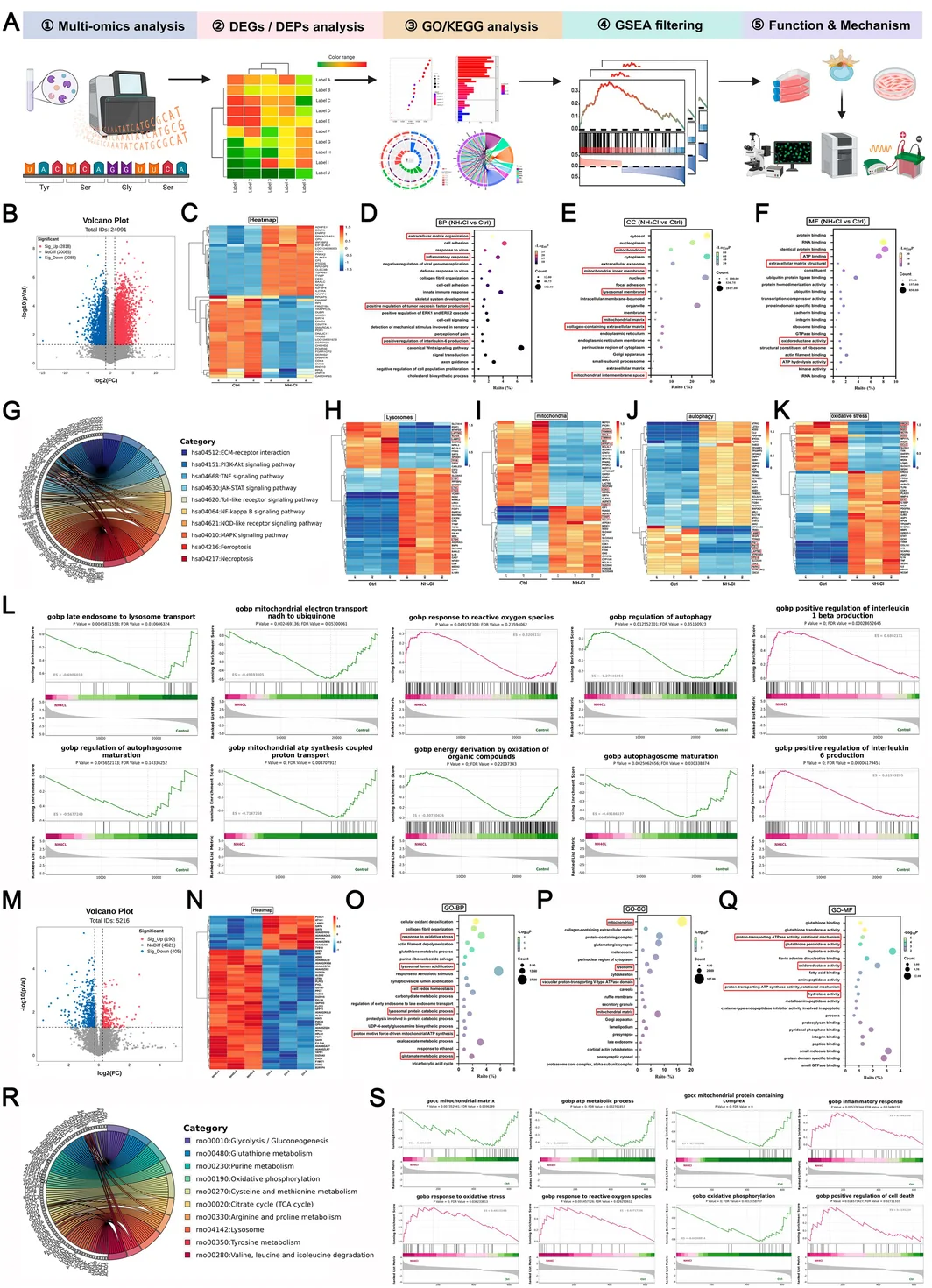
Multi‐omics analyses reveal ammonia‐induced lysosomal and mitochondrial dysfunction, autophagy suppression, and oxidative stress in NPCs. (A) Schematic workflow of integrated transcriptomic and proteomic analyses. (B) Volcano plot of differentially expressed genes (DEGs) between control and NH_4_Cl‐treated NPCs, showing up‐ and downregulated genes. (C) Heatmap of DEGs illustrating distinct transcriptional profiles in ammonia‐treated NPCs. (D–F) GO enrichment analysis for BP, CC, and MF, highlighting pathways associated with extracellular matrix organisation, inflammatory response, lysosome, mitochondrion, ATP binding, and oxidoreductase activity. (G) KEGG analysis of transcriptomic data showing significantly altered pathways in NH_4_Cl‐treated NPCs relative to control. (H–K) Heatmaps of selected gene sets related to lysosomal function, mitochondrial function, autophagy, and oxidative stress, showing coordinated dysregulation indicative of organelle dysfunction, autophagy impairment, and oxidative stress activation. (L) GSEA plots demonstrating downregulation of autophagy and mitochondrial energy metabolism pathways and upregulation of reactive oxygen species and pro‐inflammatory signalling. (M) Volcano plot of differentially expressed proteins (DEPs) between control and NH_4_Cl‐treated NPCs. (N) Heatmap of DEPs illustrating distinct proteomic profiles following ammonia exposure. (O–Q) GO enrichment analysis of DEPs for BP, CC, and MF, highlighting pathways related to lysosome, mitochondrion, oxidative stress, ATPase activity, and redox homeostasis. (R) KEGG pathway analysis of DEPs showing altered metabolic pathways relevant to NP cell homeostasis. (S) GSEA of proteomic data indicating downregulation of mitochondrial and energy metabolism pathways and upregulation of inflammatory and oxidative stress responses.

### 3.5 Ammonia Impairs Lysosomal Function in NPCs

To further investigate whether ammonia stress perturbs lysosomal homeostasis in NPCs, we performed a series of experiments to evaluate lysosomal integrity and function. NPCs were exposed to increasing concentrations of NH_4_Cl to model ammonia stress, followed by comprehensive assessment of lysosomal structure and function. Western blot analysis revealed that key lysosomal structural and functional proteins, including LAMP1, LAMP2, TRPML1, Cathepsin B, and V‐ATPase, progressively decreased with increasing ammonia exposure, whereas Galectin‐3, a marker of lysosomal membrane damage, was markedly upregulated (Figure 5A,B). TEM further demonstrated pronounced ultrastructural abnormalities in lysosomes following ammonia stress. In control cells, lysosomes exhibited typical round morphology with intact membranes and electron‐dense luminal contents. In contrast, ammonia‐treated cells displayed swollen and irregular lysosomes with disrupted membranes and leakage of luminal material, indicative of lysosomal structural damage (Figure 5C). To evaluate lysosomal acidification, LysoTracker Green staining was performed. Ammonia exposure resulted in a dose‐dependent reduction in lysosomal fluorescence intensity, suggesting impaired lysosomal acidification and functional disruption (Figure 5D,E). Consistently, a second lysosomal probe, LysoTracker Red DND‐99, revealed similar reductions in fluorescence signal under ammonia stress, further confirming compromised lysosomal acidity (Figure 5F,G). To quantitatively evaluate lysosomal pH, ratiometric LysoSensor Yellow/Blue staining was performed. Ammonia exposure induced a progressive decrease in the yellow‐to‐blue fluorescence ratio across treatment groups, indicating a dose‐dependent elevation of lysosomal pH and consequent lysosomal alkalinisation (Figure 5H,I). We next assessed lysosomal membrane integrity using acridine orange staining. Ammonia treatment led to a marked and progressive reduction in lysosomal red fluorescence, reflecting compromised lysosomal membrane integrity and increased lysosomal membrane permeabilisation (Figure 5J,K). To further examine lysosomal degradative capacity, immunofluorescence co‐localisation of LAMP1 and LC3 was analysed to evaluate autophagosome–lysosome interaction. Ammonia exposure markedly reduced LAMP1–LC3 co‐localisation, indicating impaired lysosomal participation in degradative processes (Figure 5L,M). In addition, co‐localisation of LAMP2 and CTSB was assessed to evaluate lysosomal membrane integrity. Ammonia stress markedly decreased LAMP2–CTSB co‐localisation and disrupted the punctate lysosomal distribution of CTSB, indicating CTSB release from lysosomes into the cytosol, a hallmark of lysosomal membrane permeabilisation (Figure 5N,O). Consistent with these findings, enzymatic activity assays revealed that ammonia exposure significantly decreased the activities of Cathepsin B and β‐hexosaminidase in a concentration‐dependent manner, further confirming lysosomal functional impairment (Figure 5P,Q). Finally, in vivo validation in rat intervertebral discs showed that ammonia exposure reduced the expression of LAMP1 and Cathepsin B in NP tissues, with more pronounced decreases observed in the high‐dose ammonia group (Figure 5R,S). Collectively, these findings indicate that ammonia stress compromises lysosomal integrity and function in NPCs, resulting in defective lysosomal acidification, membrane destabilisation, and impaired degradative capacity.

**FIGURE 5:**
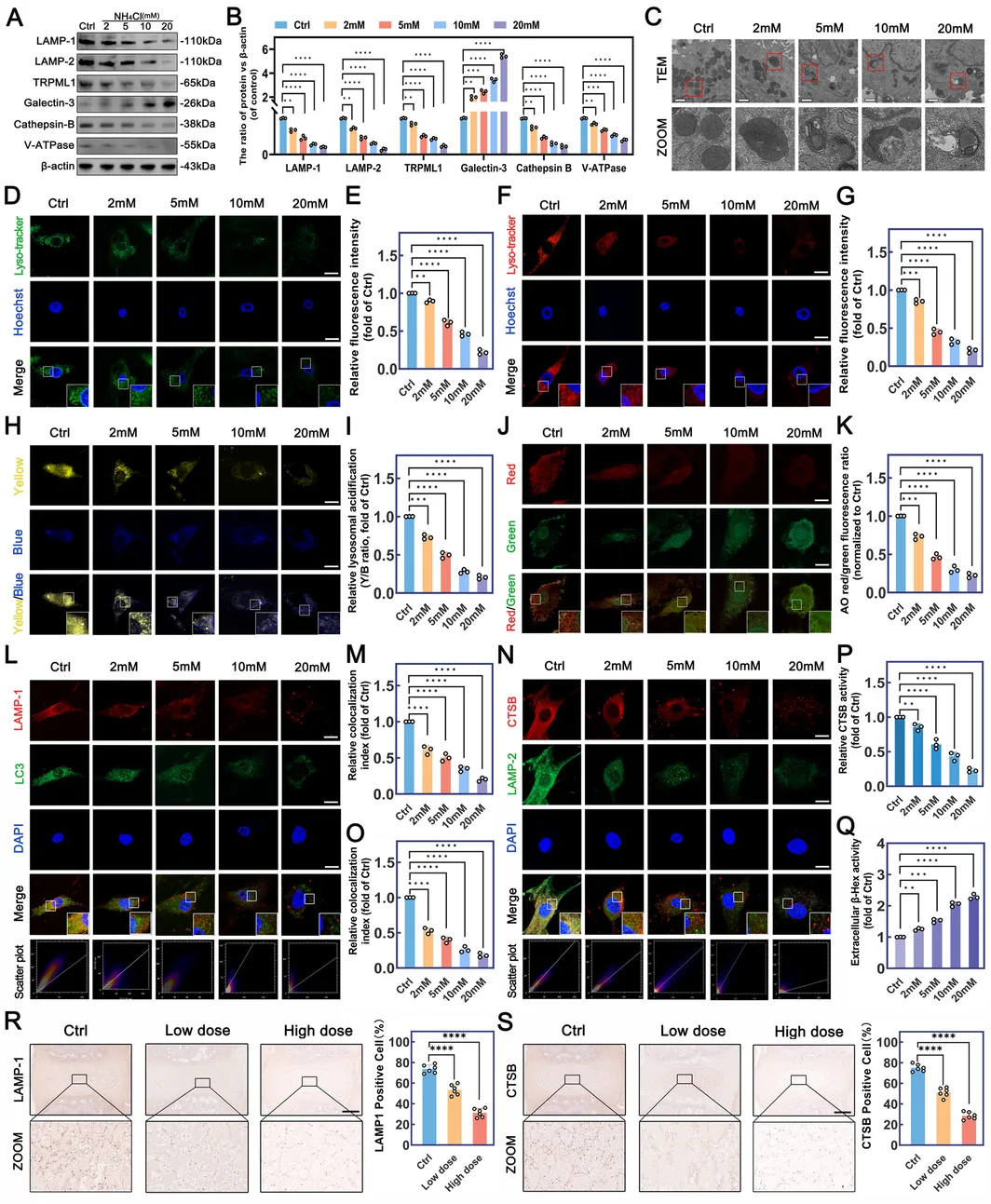
Ammonia stress disrupts lysosomal integrity and function in NPCs. (A, B) Western blot analysis and quantification of lysosomal proteins LAMP1, LAMP2, TRPML1, Cathepsin B (CTSB), V‐ATPase, and Galectin‐3 in NPCs treated with increasing concentrations of NH_4_Cl (*n* = 3). (C) Transmission electron microscopy images showing lysosomal ultrastructure in NPCs following ammonia exposure. Ammonia treatment induces swollen and structurally disrupted lysosomes with membrane damage and luminal leakage. Scale bar, 1 μm. (D, E) Representative LysoTracker Green staining images and quantification of lysosomal acidity in NPCs treated with NH_4_Cl. Ammonia exposure leads to a dose‐dependent reduction in lysosomal fluorescence intensity (scale bar, 20 μm; *n* = 3). (F, G) Representative LysoTracker Red DND‐99 staining images and quantification of fluorescence intensity in NPCs following ammonia treatment (scale bar, 20 μm; *n* = 3). (H, I) Representative ratiometric LysoSensor Yellow/Blue staining images and quantification of the yellow‐to‐blue fluorescence ratio showing lysosomal pH changes in NPCs under ammonia stress (scale bar, 20 μm; *n* = 3). (J, K) Representative acridine orange staining images and quantification of lysosomal red fluorescence intensity indicating lysosomal membrane integrity in NPCs treated with NH_4_Cl (scale bar, 20 μm; *n* = 3). (L, M) Representative immunofluorescence images and quantification of LAMP1–LC3 co‐localisation in NPCs following ammonia exposure. Scale bars, 20 μm; *n* = 3. (N, O) Representative immunofluorescence images and quantification of LAMP2–CTSB co‐localisation in NPCs after ammonia treatment. Scale bars, 20 μm; *n* = 3. (P, Q) Enzymatic activity assays showing Cathepsin B and β‐hexosaminidase activities in NPCs following ammonia exposure (*n* = 5). (R, S) Representative immunohistochemical staining and quantification of LAMP1 and CTSB expression in rat NP tissues after ammonia treatment (scale bars: 500 μm; *n* = 6). Data are presented as mean ± SD. Statistical significance was determined by one‐way ANOVA followed by Tukey's multiple comparisons test. *p < 0.05, **p < 0.01, ***p < 0.001, ****p < 0.0001; ns, not significant.

### 3.6 Ammonia Stress Disrupts Mitochondrial and Redox Homeostasis in NPCs

Based on the multi‐omics analyses in Figure 4 indicating mitochondrial dysfunction and redox imbalance in ammonia‐treated NPCs, we next investigated whether ammonia stress disrupts mitochondrial homeostasis and promotes oxidative stress. Given the central role of mitochondria in cellular redox regulation and as a major source of intracellular reactive oxygen species (ROS), mitochondrial function and oxidative stress responses were systematically evaluated. Western blot analysis demonstrated that ammonia exposure markedly altered the expression of key mitochondrial structural and functional proteins. Specifically, the levels of TOMM20, VDAC1, and ATP5A progressively decreased with increasing NH_4_Cl concentration, whereas the mitochondrial stress‐related proteins S‐OPA1, Cyt‐C, and p‐DRP1 were significantly elevated (Figure 6A,B), suggesting progressive mitochondrial structural disruption and enhanced mitochondrial fission. To further evaluate redox regulatory pathways, oxidative stress–related proteins were assessed by Western blot. The antioxidant defence components HO‐1, NQO1, SOD2, SLC7A11, and Nrf2 were progressively downregulated with increasing ammonia exposure, whereas Keap1 expression was correspondingly increased (Figure 6C,D), indicating impaired cellular antioxidant capacity and redox imbalance. Transmission electron microscopy revealed pronounced ultrastructural abnormalities in mitochondria following ammonia stress. In control NPCs, mitochondria displayed elongated morphology with intact double membranes and well‐organised cristae. In contrast, ammonia‐treated cells exhibited rounded and swollen mitochondria, marked cristae disruption or disappearance, increased electron density, and structural fragmentation, consistent with severe mitochondrial damage (Figure 6E). Mito‐Tracker staining further demonstrated that ammonia exposure significantly reduced mitochondrial abundance and disrupted mitochondrial morphology in a dose‐dependent manner, accompanied by diminished mitochondrial fluorescence intensity, indicative of compromised mitochondrial integrity and activity (Figure 6F and Figure S8A). Consistent with these findings, JC‐1 staining revealed a marked loss of mitochondrial membrane potential upon ammonia exposure, as evidenced by a decreased red/green fluorescence ratio, indicating impaired mitochondrial function (Figure 6G and Figure S8B). In line with these observations, qPCR analysis revealed that ammonia exposure dose‐dependently increased cytosolic mitochondrial DNA levels, further supporting the loss of mitochondrial integrity and the release of mitochondrial components into the cytoplasm (Figure S9A). To assess mitochondrial bioenergetic function, ATP assays further demonstrated a dose‐dependent decline in intracellular ATP levels upon ammonia exposure (Figure S9B). Seahorse XF analysis further revealed a pronounced metabolic shift in NPCs following ammonia exposure. Specifically, oxygen consumption rate (OCR) was markedly reduced, whereas extracellular acidification rate (ECAR) and proton efflux rate (PER) were significantly increased, indicating suppressed oxidative phosphorylation and enhanced glycolytic activity (Figure 6H). Quantitative analysis confirmed that key parameters of mitochondrial respiration, including basal respiration, maximal respiration, ATP production, spare respiratory capacity, and coupling efficiency, were significantly decreased, while proton leak and basal glycolysis were elevated (Figure 6I). Collectively, these findings demonstrate that ammonia stress disrupts mitochondrial bioenergetic homeostasis and drives a metabolic shift from oxidative phosphorylation towards glycolysis in NPCs. Given the central role of mitochondria in redox homeostasis, we next examined whether ammonia exposure induces oxidative stress in NPCs. Mito‐SOX staining revealed a dose‐dependent increase in mitochondrial superoxide production following ammonia treatment (Figure S10A,B). Consistently, intracellular ROS levels, as assessed by fluorescent probes, were markedly elevated with increasing ammonia concentration (Figure S11A,B). Together, these results indicate that ammonia stress induces excessive oxidative stress, further exacerbating mitochondrial dysfunction and redox imbalance in NPCs. To further clarify the temporal relationship between lysosomal and mitochondrial abnormalities during ammonia stress, we performed a refined time‐course analysis during the first 3 h of NH_4_Cl exposure. LAMP1 expression was significantly reduced as early as 0.5 h, followed by a subsequent decrease in CTSB, whereas the mitochondrial proteins TOMM20 and ATP5A remained largely unchanged during the initial phase and decreased significantly at 2 h. These findings indicate that lysosomal alterations precede detectable mitochondrial abnormalities during the early response to ammonia stress (Figure S12A,B). Finally, in vivo validation using the ammonia‐treated rat model demonstrated that the mitochondrial marker TOMM20 and the antioxidant protein SLC7A11 were progressively reduced in NP tissues with increasing ammonia exposure, further confirming mitochondrial impairment and oxidative stress in vivo (Figure 6J,K). Collectively, these findings demonstrate that ammonia stress profoundly disrupts mitochondrial and redox homeostasis in NPCs, driving mitochondrial structural and functional deterioration, bioenergetic failure, and uncontrolled ROS accumulation.

**FIGURE 6:**
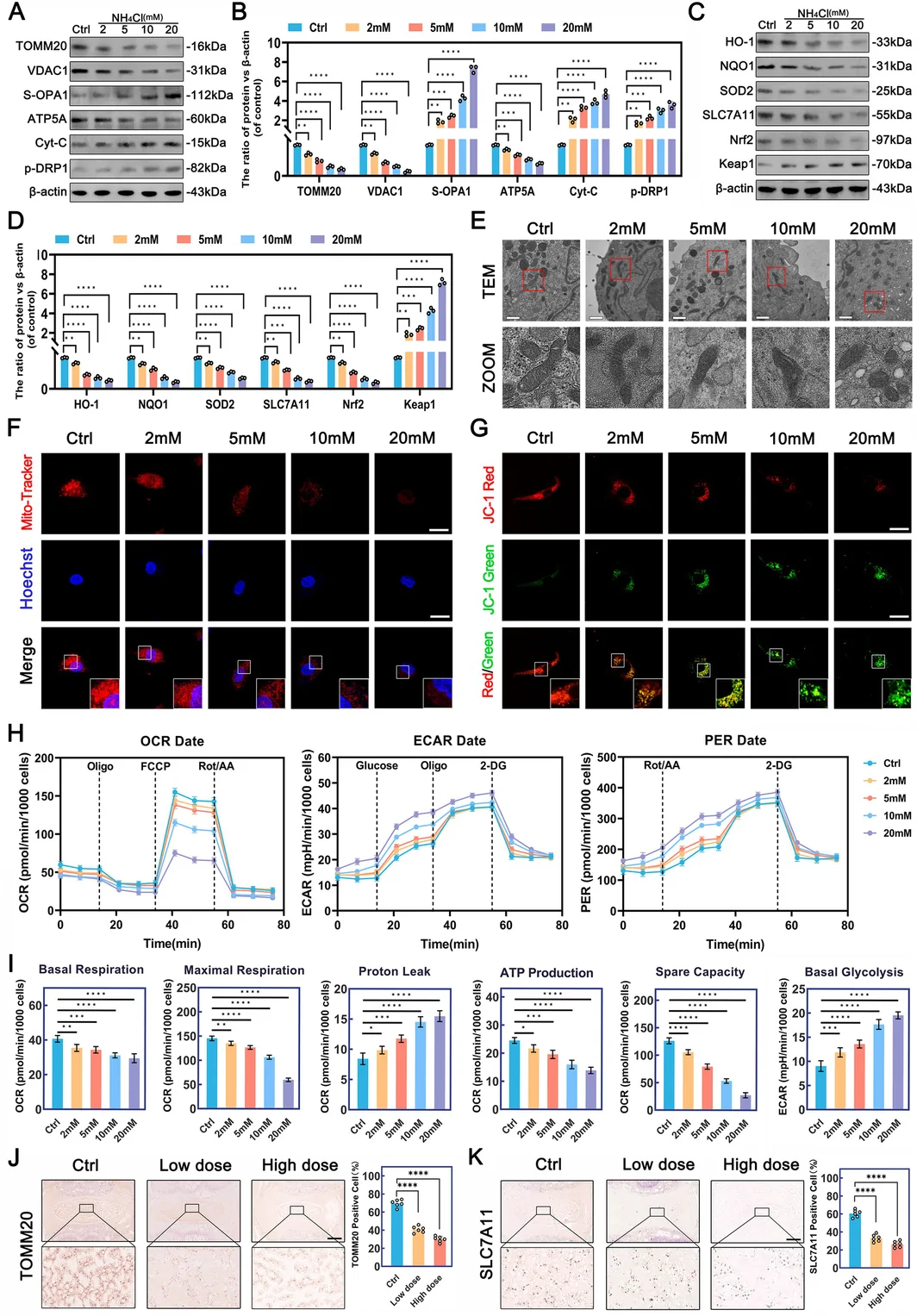
Ammonia stress disrupts mitochondrial and redox homeostasis in NPCs. (A, B) Western blot analysis and quantitative evaluation of mitochondrial structural and functional proteins TOMM20, VDAC1, ATP5A, S‐OPA1, Cyt‐C, and p‐DRP1 in NPCs treated with increasing concentrations of NH_4_Cl (*n* = 3). (C, D) Western blot analysis and corresponding quantification of oxidative stress–related proteins HO‐1, NQO1, SOD2, SLC7A11, Nrf2, and Keap1 in NPCs following ammonia exposure (*n* = 3). (E) Representative TEM images showing mitochondrial ultrastructure in control and ammonia‐treated NPCs. Scale bar = 1 μm. (F) Representative fluorescence images of Mito‐Tracker staining showing mitochondrial morphology and distribution in NPCs under different NH_4_Cl treatments (scale bar = 20 μm, *n* = 3). (G) Mitochondrial membrane potential detected by JC‐1 staining, with representative fluorescence images showing red and green fluorescence signals in NPCs following ammonia treatment (scale bar = 20 μm, *n* = 3). (H) Seahorse XF metabolic flux analysis showing the oxygen consumption rate (OCR), extracellular acidification rate (ECAR), and proton efflux rate (PER) profiles of NPCs treated with increasing concentrations of NH_4_Cl (*n* = 3). (I) Quantitative analysis of basal respiration, maximal respiration, proton leak, ATP production, spare respiratory capacity, and basal glycolysis derived from the Seahorse XF measurements. (J, K) Immunohistochemical staining and quantitative analysis of TOMM20 and SLC7A11 expression in NP tissues from the ammonia‐treated rat model. Scale bar = 500 μm, *n* = 6. All data are expressed as mean ± SD. Statistical significance is indicated as follows: *p < 0.05, **p < 0.01, ***p < 0.001, ****p < 0.0001; ns denotes a comparison that was not statistically significant.

### 3.7 Ammonia Stress Disrupts Mitophagy Homeostasis in NPCs

The multi‐omics analyses in Figure 4 revealed suppression of autophagy accompanied by mitochondrial and lysosomal dysfunction under ammonia stress, prompting us to further investigate whether ammonia exposure disrupts mitophagy, a specialised autophagic pathway responsible for mitochondrial quality control through lysosome‐dependent degradation. To evaluate the effect of ammonia stress on mitophagy in NPCs, the expression of key autophagy‐ and mitophagy‐related proteins was first examined by Western blot. As shown in Figure 7A,B, increasing NH_4_Cl exposure progressively reduced the expression of PINK1, Parkin, Atg5, and Atg12, accompanied by a gradual decrease in the LC3‐II/I ratio, whereas the autophagy adaptor p62 accumulated in a concentration‐dependent manner. Collectively, these findings indicate that ammonia stress markedly suppresses mitophagy in NPCs. To visualise autophagic structures, TEM was performed to examine the ultrastructural features of autophagosomes and autolysosomes. As shown in Figure 7C, control NPCs displayed typical double‐membrane autophagosomes and autolysosomal structures containing partially degraded cytoplasmic components. In contrast, ammonia exposure markedly reduced autophagic vesicles and induced structural abnormalities. Notably, the proportion of autolysosomes relative to total autophagic vesicles progressively decreased with increasing NH_4_Cl concentration, suggesting impaired autophagic flux. Given the central role of PINK1–Parkin signalling in initiating mitophagy through the recruitment of Parkin to damaged mitochondria, we next assessed the colocalisation of Parkin and the mitochondrial outer membrane marker TOMM20 by immunofluorescence. As shown in Figure 7D,E, ammonia exposure progressively reduced the colocalisation between Parkin and TOMM20, indicating impaired recruitment of Parkin to mitochondria and suppression of PINK1–Parkin–mediated mitophagy initiation. To assess autophagosome–lysosome fusion, the colocalisation of LC3 with the lysosomal marker LAMP1 was analysed by immunofluorescence. As shown in Figure 7F,G, ammonia stress dose‐dependently reduced LC3–LAMP1 colocalisation, indicating impaired autophagosome–lysosome fusion and defective autophagic degradation. Autophagic flux was further assessed using the GFP‐RFP‐LC3 tandem fluorescent reporter. As shown in Figure 7H,I, ammonia exposure markedly reduced the number of autolysosomal puncta and decreased overall autophagic flux in NPCs in a concentration‐dependent manner, further supporting inhibition of autophagy progression. To directly assess mitophagy activity, the Mito‐Keima fluorescent probe was used to analyse mitophagy levels in NPCs across different treatment groups. Quantification of the red‐to‐green fluorescence ratio revealed that ammonia stress significantly suppressed mitophagy in a dose‐dependent manner (Figure 7J,K). Consistently, dual staining with Mito‐Tracker and Lyso‐Tracker demonstrated reduced mitochondrial–lysosomal colocalisation following ammonia exposure (Figure 7L,M), further confirming impaired mitochondrial delivery to lysosomes. To further delineate how ammonia stress suppresses mitophagy, we employed the lysosomal inhibitor bafilomycin A1 (BafA1) to dissect the effects of ammonia exposure on different stages of the autophagic process. NPCs were treated with Control, BafA1, NH_4_Cl, or NH_4_Cl + BafA1. Immunofluorescence analysis of LC3 and p62 was performed to evaluate autophagy activation and degradation. As shown in Figure 7N,O, BafA1 treatment markedly increased LC3 accumulation compared with the control group, whereas NH_4_Cl exposure significantly reduced LC3 levels, indicating suppression of autophagy activation under ammonia stress. In parallel, p62 levels were markedly elevated in the NH_4_Cl and NH_4_Cl + BafA1 groups, suggesting impaired autophagic degradation. Collectively, these findings suggest that ammonia stress suppresses mitophagy by simultaneously inhibiting autophagy activation and impairing autophagic degradation. Consistent with the in vitro findings, in vivo validation using the ammonia‐treated rat model demonstrated that the expression of PINK1 and LC3 in NP tissues was markedly reduced following ammonia exposure, with more pronounced downregulation observed in the high‐dose group (Figure 7P–S). Collectively, these findings demonstrate that ammonia stress profoundly suppresses mitophagy in NPCs by inhibiting PINK1–Parkin–mediated mitophagy initiation and impairing lysosome‐dependent autophagic degradation, thereby contributing to mitochondrial quality‐control failure under ammonia stress.

**FIGURE 7:**
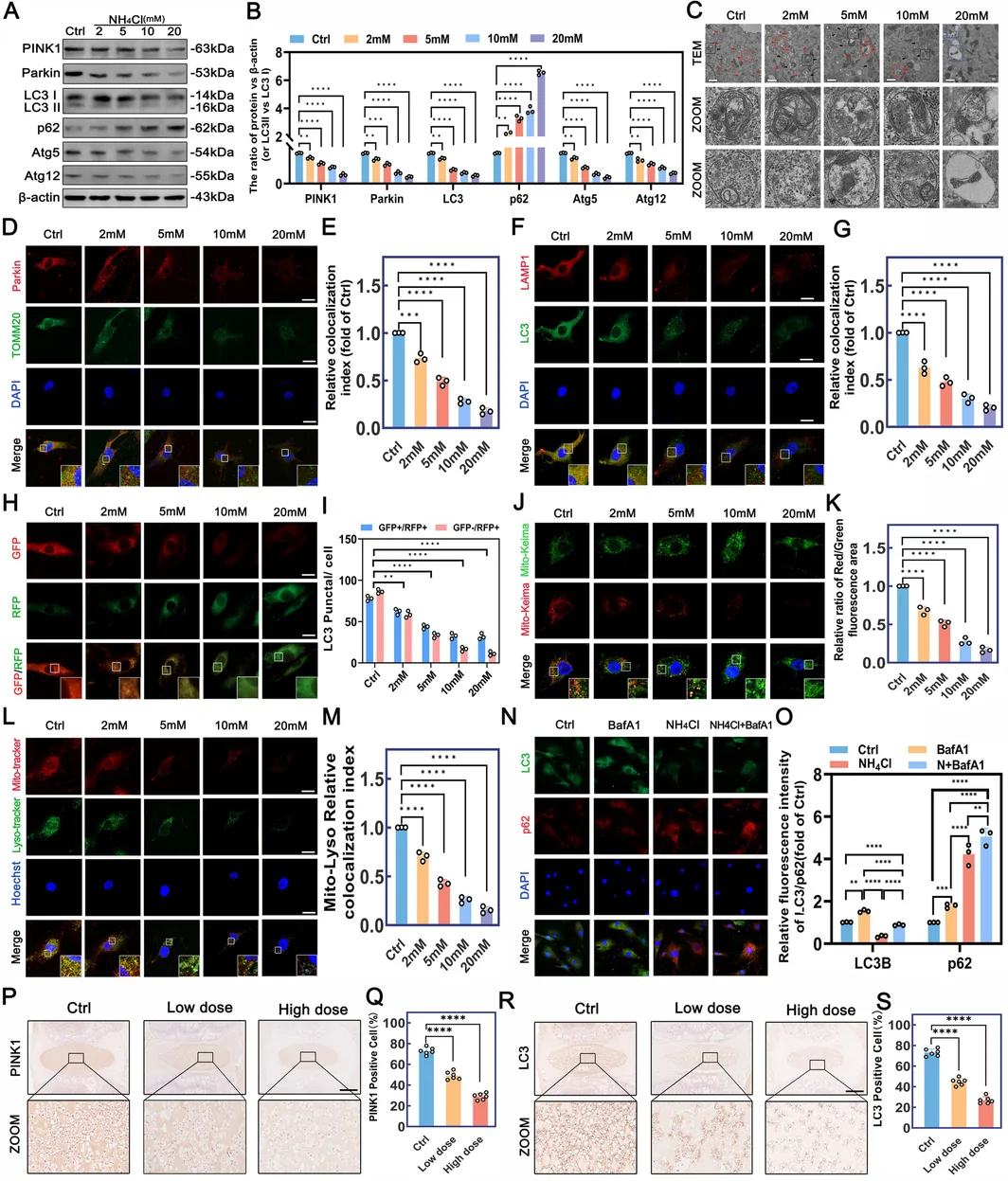
Ammonia stress disrupts mitophagy homeostasis in NPCs. (A, B) Western blot analysis and quantitative evaluation of mitophagy‐ and autophagy‐related proteins, including PINK1, Parkin, Atg5, Atg12, LC3‐II/I, and p62, in NPCs treated with increasing concentrations of NH_4_Cl (*n* = 3). (C) TEM images showing ultrastructural features of autophagosomes and autolysosomes in NPCs following ammonia exposure. Scale bar = 1 μm. (D, E) Representative immunofluorescence images and quantitative analysis of the colocalisation of Parkin with the mitochondrial outer membrane marker TOMM20 in NPCs under ammonia stress, indicating reduced recruitment of Parkin to mitochondria (scale bars: 20 μm; *n* = 3). (F, G) Representative immunofluorescence images and quantitative analysis of the colocalisation of LC3 with the lysosomal marker LAMP1 in NPCs treated with NH_4_Cl, reflecting impaired autophagosome–lysosome fusion under ammonia stress. Scale bar = 20 μm; *n* = 3. (H, I) Representative images and quantitative analysis of autophagic flux in NPCs expressing the GFP–RFP–LC3 tandem fluorescent reporter under ammonia exposure. Ammonia stress markedly reduced autophagic flux (scale bars: 20 μm; *n* = 3). (J, K) Representative images and quantitative analysis of mitophagy activity detected using the Mito‐Keima fluorescent probe in NPCs under different ammonia treatments, showing a dose‐dependent reduction in the red/green fluorescence ratio (scale bars: 20 μm; *n* = 3). (L, M) Representative images and quantitative analysis of mitochondrial–lysosomal colocalisation in NPCs stained with Mito‐Tracker and Lyso‐Tracker, demonstrating reduced mitochondrial delivery to lysosomes following ammonia exposure (scale bars: 20 μm; *n* = 3). (N, O) Representative immunofluorescence images and quantitative analysis of LC3 and p62 in NPCs treated with Control, BafA1, NH_4_Cl, or NH_4_Cl + BafA1, indicating suppression of autophagy activation and impaired autophagic degradation under ammonia stress (scale bars: 25 μm; *n* = 3). (P, Q) Representative immunohistochemical images and quantitative analysis of PINK1 expression in NP tissues from rats in different groups. Scale bar = 500 μm; *n* = 6. (R, S) Representative immunohistochemical images and quantitative analysis of LC3 expression in NP tissues from rats in different groups (scale bars: 500 μm; *n* = 6). Data are presented as mean ± SD. Statistical significance is indicated as follows: *p < 0.05, **p < 0.01, ***p < 0.001, ****p < 0.0001; ns, not significant.

### 3.8 Ammonia‐Induced NP Cell Death Plays an Important Role in the Pathogenesis of IVDD

Given that mitochondrial dysfunction and oxidative stress are common upstream events driving multiple forms of programmed cell death, we next investigated whether ammonia‐induced NP cell death corresponds to known death modalities, including ferroptosis, pyroptosis, necroptosis, or apoptosis. To this end, we first examined the expression of ferroptosis‐related proteins under increasing ammonia stress. Western blot analysis revealed a progressive decrease in GPX4, FTH1, and xCT expression accompanied by an increase in LPCAT3 expression (Figure 8A and Figure S13A). We then assessed the activation of pyroptosis‐associated signalling and observed that the levels of NLRP3, ASC, Caspase‐1, and GSDMD‐N increased in a dose‐dependent manner following ammonia exposure (Figure 8B and Figure S13B). Similarly, ammonia stress markedly elevated the expression of necroptosis‐related proteins, including p‐RIPK1, p‐RIPK3, and p‐MLKL (Figure 8C and Figure S13C). To further determine whether modulation of ammonia metabolism contributes to the protective effects of GLS1 inhibition, we examined the expression of the ammonia‐metabolising enzymes GLUL and GLUD1 under CB839 rescue conditions. NH_4_Cl exposure markedly reduced GLUL expression while increasing GLUD1 expression. CB839 treatment partially restored GLUL expression and attenuated the NH_4_Cl‐induced upregulation of GLUD1, whereas CB839 alone exerted no marked effect under basal conditions (Figure S14A,B). These results suggest that CB839 may partially restore ammonia‐metabolic homeostasis and GLUL‐associated ammonia detoxification capacity. Although ammonia exposure was accompanied by the activation of multiple programmed cell‐death pathways, whether ammonia‐induced NP cell death could be attributed to any single canonical modality remained unclear. We therefore performed rescue experiments using inhibitors of ferroptosis, pyroptosis, necroptosis, and apoptosis, together with CB839 to suppress glutaminase‐dependent ammonia production. Western blot analysis showed that inhibition of ferroptosis using Fer‐1 partially restored GPX4 and FTH1 expression while reducing LPCAT3 and ACSL4 levels under ammonia stress; however, suppression of ammonia production by CB839 exerted a markedly stronger rescue effect on these ferroptosis‐related markers (Figure 8D and Figure S13D). A similar pattern was observed for pyroptosis‐related signalling. Although MCC950 moderately attenuated the ammonia‐induced upregulation of NLRP3, ASC, Caspase‐1, and GSDMD‐N, CB839 more effectively suppressed the activation of these proteins (Figure 8E and Figure S15A). Consistently, inhibition of necroptosis with Nec‐1 partially reduced the ammonia‐induced increases in p‐RIPK1, p‐RIPK3, and p‐MLKL, whereas CB839 produced a more pronounced inhibitory effect (Figure 8F and Figure S15B). Likewise, blockade of apoptosis with Z‐VAD‐FMK moderately attenuated the ammonia‐induced upregulation of Bax, cleaved‐caspase‐3, and cleaved‐caspase‐7 and restored Bcl‐2 expression, yet CB839 again exhibited a stronger protective effect (Figure 8G and Figure S15C). Functional assays further supported these observations. Cell viability analysis showed that inhibition of individual programmed cell death pathways using Fer‐1, MCC950, Nec‐1, or Z‐VAD‐FMK failed to effectively rescue ammonia‐induced NP cell death, whereas CB839 markedly restored cell viability (Figure 8H).

**FIGURE 8:**
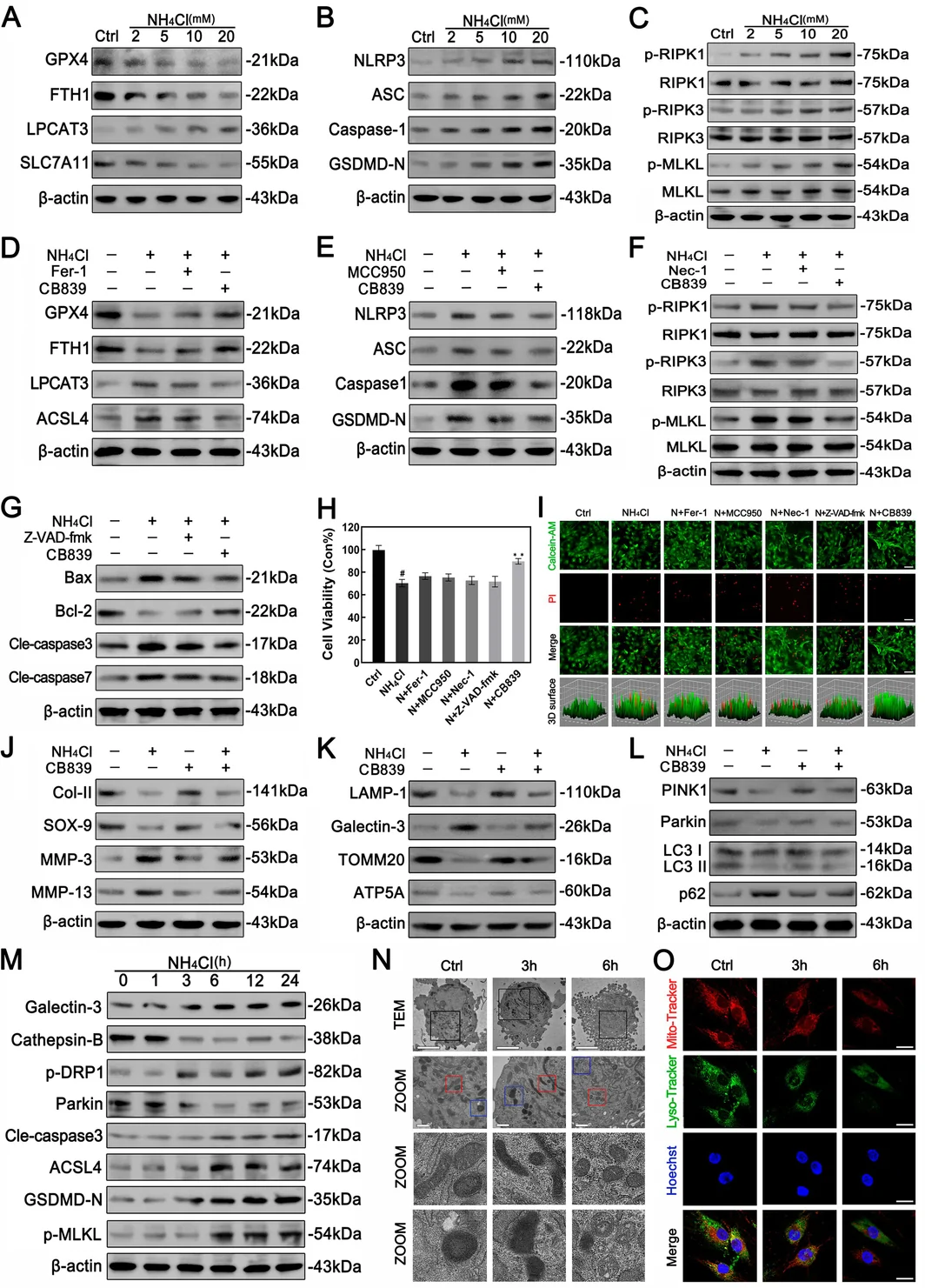
Ammonia‐induced NP cell death plays a critical role in IVDD. (A) Western blot analysis of ferroptosis‐related proteins (GPX4, FTH1, SLC7A11, LPCAT3) under increasing ammonia stress (*n* = 3). (B) Western blot analysis of pyroptosis‐associated proteins (NLRP3, ASC, Caspase‐1, GSDMD‐N) in NPCs treated with ammonia (*n* = 3). (C) Western blot analysis of necroptosis markers (p‐RIPK1, p‐RIPK3, p‐MLKL) under ammonia stress (*n* = 3). (D) Western blot detection of ferroptosis markers in NPCs treated with ammonia alone or combined with Fer‐1 or CB839 (*n* = 3). (E) Western blot detection of pyroptosis‐associated proteins in NPCs treated with ammonia alone or combined with MCC950 or CB839 (*n* = 3). (F) Western blot analysis of necroptosis markers in NPCs treated with ammonia alone or combined with Nec‐1 or CB839 (*n* = 3). (G) Western blot analysis of apoptosis markers (Bax, Bcl‐2, cleaved‐caspase‐3, cleaved‐caspase‐7) in NPCs treated with ammonia alone or combined with Z‐VAD‐FMK or CB839 (*n* = 3). (H) CCK‐8 assay to measure NP cell viability under different ammonia stress and inhibitor treatments (*n* = 5). (I) Calcein‐AM staining to evaluate NP cell survival under ammonia stress and pharmacological inhibitor treatments (scale bars: 50 μm; *n* = 3). (J) Western blot analysis of NP cell phenotype markers (Col‐II, SOX9, MMP‐3, MMP‐13) after ammonia exposure with or without CB839 treatment (*n* = 3). (K) Western blot detection of lysosomal and mitochondrial proteins (LAMP‐1, TOMM20, ATP5A, Galectin‐3) in ammonia‐treated NPCs with or without CB839 (*n* = 3). (L) Western blot analysis of mitophagy‐related proteins (PINK1, Parkin, LC3‐II/I, p62) under ammonia stress with or without CB839 treatment (*n* = 3). (M) Time‐course Western blot analysis of NPCs treated with NH_4_Cl, detecting lysosomal and mitochondrial markers (Galectin‐3, CTSB, p‐DRP1, Parkin) and canonical cell death markers (cleaved‐caspase‐3, ACSL4, GSDMD‐N, p‐MLKL). (N) TEM of NPCs at 0, 3, and 6 h of ammonia treatment to assess lysosomal and mitochondrial ultrastructural changes. (O) MitoTracker and LysoTracker co‐staining of NPCs at 0, 3, and 6 h of ammonia treatment to visualise mitochondrial and lysosomal integrity (*n* = 3). Data are presented as mean ± SD. Statistical significance is indicated as follows: *p < 0.05, **p < 0.01, ***p < 0.001, ****p < 0.0001, #*p* < 0.05; ns, not significant.

Similar results were obtained using Calcein‐AM staining to evaluate cell survival, which closely mirrored the findings of the CCK‐8 assay. Notably, inhibition of ferroptosis with Fer‐1 partially attenuated ammonia‐induced NP cell death, whereas inhibitors targeting pyroptosis, necroptosis, or apoptosis exerted minimal protective effects. However, the rescue effect of Fer‐1 remained markedly weaker than that achieved by CB839 (Figure 8I and Figure S15D). These findings suggest that ammonia‐induced NP cell death may share certain mechanistic features with ferroptosis, yet is not fully attributable to this canonical death modality. Consistent with these findings, EdU staining further demonstrated similar trends in NP cell proliferation across different treatment groups (Figure S16A,B). Together, these findings indicate that ammonia‐induced NP cell death cannot be fully explained by canonical ferroptosis, pyroptosis, necroptosis, or apoptosis, suggesting that ammonia stress may trigger a distinct form of cell death while secondarily activating multiple programmed cell death pathways. To further determine whether ammonia accumulation is a central driver of the pathological changes observed in NPCs, we next performed rescue experiments using CB839 to suppress ammonia production. As shown in Figure 8J and Figure S17A, CB839 markedly reversed the ammonia‐induced downregulation of Col‐II and SOX9 as well as the upregulation of MMP‐3 and MMP‐13, indicating significant restoration of the NP cell phenotype. Furthermore, CB839 alleviated ammonia‐induced lysosomal and mitochondrial abnormalities, as evidenced by the restoration of LAMP‐1, TOMM20, and ATP5A expression and the reduction of Galectin‐3 levels (Figure 8K and Figure S17B). Consistently, CB839 also rescued the impairment of mitophagy, reversing the ammonia‐induced decreases in PINK1, Parkin, and LC3‐II/I while reducing p62 accumulation (Figure 8L and Figure S17C). These findings further support a central role of ammonia stress in driving NP cell degeneration and mitochondrial quality‐control failure. To determine the temporal relationship between ammonia‐induced cellular alterations and canonical programmed cell death pathways, we next performed a time‐course analysis following NH_4_Cl treatment. Western blot analysis showed that early markers of lysosomal and mitochondrial dysfunction, including Galectin‐3 and p‐DRP1, were significantly increased as early as 3 h after ammonia exposure, whereas CTSB and Parkin expression began to decline at this time point. In contrast, markers of apoptosis, ferroptosis, pyroptosis, and necroptosis, including cleaved‐caspase‐3, ACSL4, GSDMD‐N, and p‐MLKL, were not significantly elevated until 6 h of ammonia treatment, indicating that the cellular death features induced by ammonia stress arise earlier than the activation of these canonical programmed cell death pathways (Figure 8M and Figure S18A). TEM further confirmed these observations. Structural abnormalities of lysosomes and mitochondria were already detectable at 3 h after ammonia exposure and became more pronounced at 6 h, consistent with the early molecular alterations observed by Western blot (Figure 8N). Similarly, Mito‐Tracker and Lyso‐Tracker co‐staining revealed corresponding mitochondrial and lysosomal damage at these time points, further supporting that ammonia‐induced lysosomal and mitochondrial dysfunction precedes the activation of canonical programmed cell death pathways (Figure 8O and Figure S18B). Collectively, these findings indicate that ammonia stress triggers a distinct form of NP cell death characterised by early lysosomal damage, mitochondrial dysfunction, and impaired mitophagy, which acts as an upstream pathological driver that precedes and potentially initiates multiple programmed cell death pathways during IVDD.

### 3.9 Targeting Ammonia Stress Alleviates IVDD In Vivo

To validate the role of ammonia stress in IVDD in vivo, we established a rat tail disc degeneration model and performed pharmacological interventions targeting ammonia production or oxidative stress. The experimental workflow and grouping strategy are illustrated in Figure 9A, including the control group, ammonia overload model group, CB839‐treated group, model combined with CB839, and model combined with the antioxidant NAC. In parallel, histological assessment of major organs was performed to exclude potential systemic toxicity, thereby ensuring the reliability of the ammonia stress model (Figure S19). MRI was performed to evaluate disc degeneration in different groups. As shown in Figure 9B, the model group displayed markedly reduced T2‐weighted signal intensity, disc height, and disc volume compared with the control group. Administration of CB839 alone produced imaging features largely comparable to those of the control group. Notably, CB839 treatment substantially alleviated the degenerative changes observed in the model group, whereas NAC treatment also improved disc morphology but to a lesser extent than CB839. Quantitative analysis based on Pfirrmann grading further confirmed these observations (Figure 9M). Histological staining was subsequently performed to further assess structural changes in the intervertebral discs. Haematoxylin and eosin (H&E) staining revealed marked disruption of disc architecture in the model group, characterised by loss of NP structure and disorganisation of the annulus fibrosus (Figure 9C). Safranin O–Fast Green staining further showed a pronounced reduction in proteoglycan content. These alterations were substantially alleviated in the CB839‐treated model group, whereas NAC produced only moderate improvement. Histological scoring further supported these observations (Figure 9N). Similar trends were observed in Alcian blue and Masson staining (Figure 9D). Alcian blue staining demonstrated a marked loss of acidic glycosaminoglycans in the model group, while Masson staining revealed increased fibrotic deposition within the disc tissue. These degenerative changes were significantly mitigated by CB839 treatment and only partially improved by NAC. To further evaluate extracellular matrix metabolism, immunohistochemical staining was performed for matrix synthesis and degradation markers. Aggrecan staining showed strong expression in the control and CB839 groups but was markedly reduced in the model group (Figure 9E,O). This reduction was substantially restored by CB839 treatment, whereas NAC produced only partial recovery. A similar pattern was observed for Col‐II expression (Figure 9F,P). In contrast, MMP‐3 staining was markedly elevated in the model group and was significantly suppressed by CB839 treatment, while NAC treatment resulted in only moderate attenuation (Figure 9G,Q). We next examined key enzymes involved in ammonia metabolism. Immunohistochemical staining showed that GLUD1 expression was markedly increased in the model group, and this increase was substantially attenuated by CB839 treatment, whereas NAC produced only partial improvement (Figure 9H,R). A similar pattern was observed for GLS1 expression (Figure 9I,S). In contrast, GLUL staining was markedly reduced in the model group and was largely restored by CB839 treatment, while NAC exhibited a weaker effect (Figure 9J,T). These findings highlight a critical role of ammonia metabolic homeostasis in the progression of IVDD. Given the central role of lysosomal and mitochondrial dysfunction observed in our in vitro experiments, we next assessed the expression of related markers by immunofluorescence staining. As shown in Figure 9K and Figure S20A, LAMP‐2 and CTSB staining was markedly reduced in the model group. CB839 treatment largely restored their expression, whereas NAC treatment produced only partial recovery. A similar pattern was observed for mitochondrial markers TOMM20 and COX IV (Figure 9L and Figure S20B), with CB839 showing a more pronounced rescue effect than NAC. These findings suggest that ammonia overload in vivo is associated with lysosomal and mitochondrial dysfunction, which can be effectively alleviated by inhibition of ammonia production. Western blot analysis of NP tissues further supported these observations. As shown in Figure 9U,V, the model group exhibited reduced expression of the matrix synthesis markers Col‐II and SOX9 together with increased expression of the matrix degradation enzymes MMP‐3 and MMP‐13. These alterations were significantly reversed by CB839 treatment, while NAC showed a comparatively weaker protective effect. Finally, behavioural assessments were performed to evaluate pain‐related functional outcomes. Compared with the control group, the model group showed markedly reduced pressure tolerance, decreased travel distance, shortened activity time, and lower maximal speed. Treatment with NAC partially improved these behavioural parameters, whereas CB839 produced a more pronounced improvement (Figure 9W–Z), consistent with the imaging and histological findings. Collectively, these results demonstrate that pharmacological inhibition of ammonia production effectively alleviates IVDD, restores matrix homeostasis, and improves functional outcomes in vivo, further supporting a central role of ammonia stress in driving disc degeneration.

**FIGURE 9:**
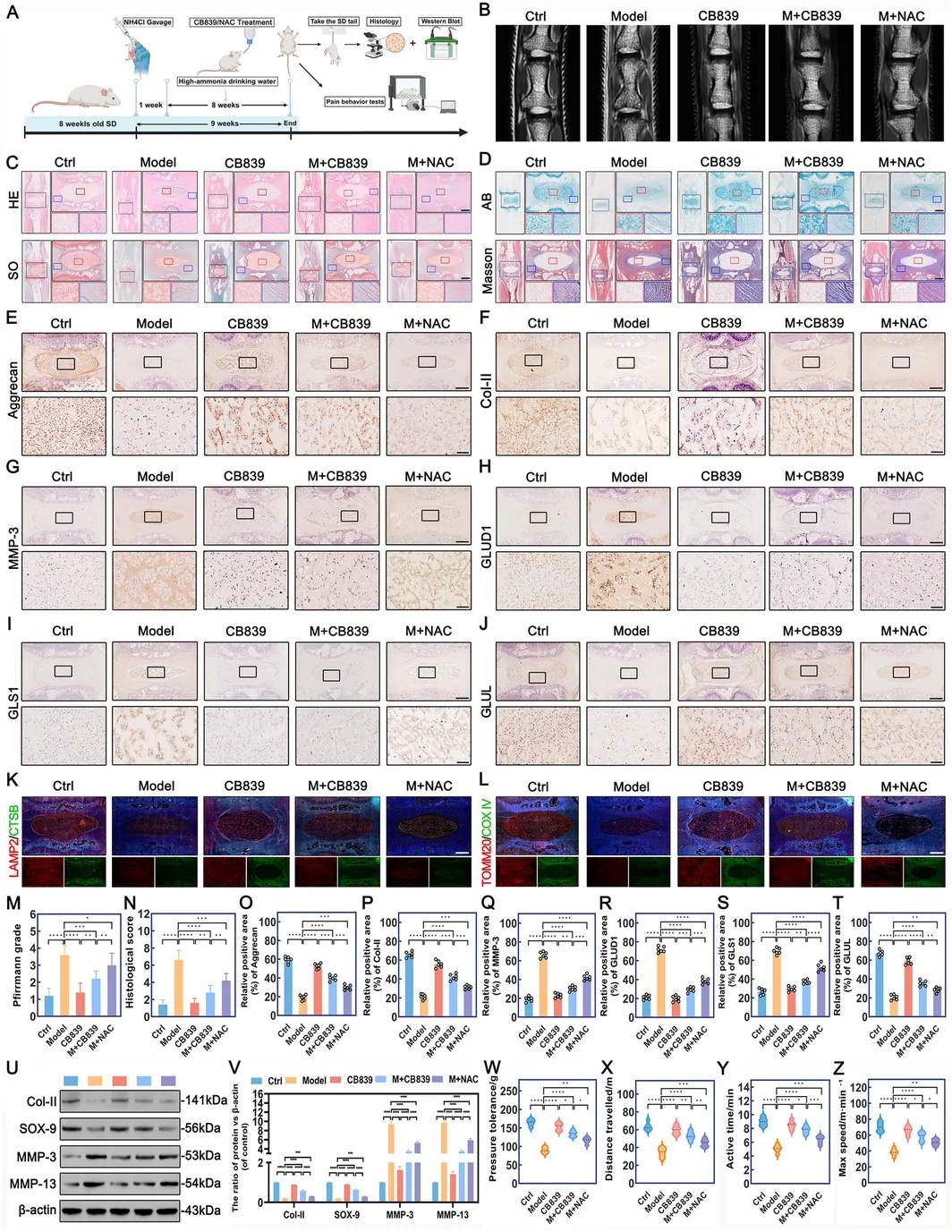
Suppressing ammonia stress protects against IVDD in vivo. (A) Schematic diagram illustrating the experimental design and grouping strategy for the in vivo study. (B) Representative T2‐weighted MRI of rat tail intervertebral discs from different experimental groups (*n* = 6). (C) Representative images of H&E staining and Safranin O–Fast Green staining of rat intervertebral disc tissues. (scale bars: 500 μm; *n* = 6). (D) Representative images of Alcian blue staining and Masson staining of rat intervertebral disc tissues (scale bars: 500 μm; *n* = 6). (E–G) Immunohistochemical staining of Aggrecan, Col‐II and MMP‐3 in rat intervertebral disc tissues (scale bars: 500 μm; *n* = 6). (H–J) Immunohistochemical staining of GLUD1, GLS1 and GLUL in rat intervertebral disc tissues (scale bars: 500 μm; *n* = 6). (K) Immunofluorescence staining of LAMP‐2 and CTSB in rat intervertebral disc tissues (scale bars: 500 μm; *n* = 6). (L) Immunofluorescence staining of TOMM20 and COX IV in rat intervertebral disc tissues (scale bars: 500 μm; *n* = 6). (M) Quantitative analysis of Pfirrmann grading based on MRI evaluation. (N) Histological scoring of IVDD. (O–T) Quantitative analysis of immunohistochemical staining for Aggrecan, Col‐II, MMP‐3, GLUD1, GLS1, and GLUL. (U, V) Western blot analysis and corresponding quantification of Col‐II, SOX9, MMP‐3, and MMP‐13 expression in rat NP tissues. (W–Z) Behavioural assessments including pressure tolerance test and locomotor activity parameters (total distance travelled, activity time, and maximum speed). Statistical significance was determined by one‐way ANOVA followed by Tukey's multiple comparisons test. *p < 0.05, **p < 0.01, ***p < 0.001, ****p < 0.0001; ns, not significant.

## 4 Discussion

In this study, we uncover a previously unrecognised role of ammonia in the pathogenesis of IVDD. While the disc microenvironment has long been characterised by acidity and metabolic imbalance, our findings extend this paradigm by identifying ammonia accumulation as a critical yet overlooked factor associated with IVDD. By integrating clinical observations with mechanistic analyses, our findings indicate that ammonia stress promotes degenerative phenotypes in NPCs and elicits a non‐canonical, metabolite‐driven cell‐death process consistent with AICD. Mechanistically, AICD is characterised by coordinated disruption of lysosomal integrity and mitochondrial function, coupled with oxidative stress and impaired mitophagy, thereby reflecting a breakdown of lysosome–mitochondria crosstalk under ammonia stress. These findings not only expand the current understanding of metabolic stress in IVDD but also establish a conceptual framework linking nitrogen metabolism dysregulation to disc degeneration.

IVDD is characterised by progressive structural and cellular deterioration within a metabolically constrained microenvironment [42, 43]. Despite extensive efforts to elucidate the pathogenesis of IVDD, effective disease‐modifying therapies remain lacking, largely due to an incomplete understanding of the metabolic landscape within the disc microenvironment. Current mechanistic frameworks have predominantly centred on inflammation, extracellular matrix degradation, and lactate‐driven acidosis as key contributors to disc degeneration [44, 45, 46]. While these paradigms have yielded important insights, they largely emphasise carbon metabolism and acidic stress, leaving other fundamental metabolic dimensions insufficiently explored. In particular, nitrogen metabolism, a critical determinant of cellular homeostasis, has been largely overlooked in the context of IVDD, despite the diffusion‐restricted microenvironment of the intervertebral disc that favours accumulation of metabolic byproducts. Ammonia, a central intermediate of nitrogen metabolism, is well recognised for its cytotoxic potential in diverse pathological conditions. However, its role in IVDD has not been systematically investigated. In the present study, through an integrated strategy combining clinical cohort analysis, human tissue characterisation, metabolomic profiling, and complementary in vivo and in vitro models, we provide converging evidence that ammonia accumulation is closely associated with disc degeneration and contributes to disease progression. Notably, our findings demonstrate that elevated ammonia levels are not only correlated with disease severity but also recapitulate key degenerative features across experimental systems, including disruption of tissue architecture and impairment of NP cell homeostasis. Furthermore, experimental modulation of ammonia exposure altered degenerative phenotypes, supporting a contributory role for ammonia‐related metabolic stress in disc pathology. This multi‐level evidence supports a model in which ammonia is not merely a byproduct of metabolic imbalance but a functional driver of pathological changes, bridging the gap between metabolic dysregulation and cellular dysfunction in IVDD. Importantly, these findings extend the current understanding of IVDD beyond a predominantly carbon metabolism–centred paradigm towards a more comprehensive framework incorporating nitrogen metabolic dysregulation. Mechanistically, the high membrane permeability of ammonia and its capacity to disrupt intracellular pH homeostasis suggest that it may affect multiple organelles, including lysosomes and mitochondria, thereby amplifying cellular stress under limited clearance. Consistent with this notion, our observations reveal coordinated alterations in subcellular homeostasis in response to ammonia stress, indicating a broader impact on intracellular quality control systems. In this context, ammonia emerges not only as a biomarker of metabolic disturbance but also as a mediator and amplifier of disease progression. Collectively, our results redefine the metabolic landscape of IVDD and establish ammonia accumulation as a previously underappreciated metabolic liability that drives disease progression, with significant pathophysiological and therapeutic implications and potential for metabolism‐targeted interventions.

PCD is widely implicated in the pathogenesis of IVDD, with apoptosis, pyroptosis, necroptosis, and ferroptosis collectively contributing to the progressive loss of NPCs and tissue degeneration [47, 48, 49]. These well‐characterised modalities have established a foundational framework for understanding cell fate regulation within the disc microenvironment. However, increasing evidence suggests that metabolite‐induced stress may trigger cell death programs that are not fully encompassed by canonical PCD classifications [50, 51, 52]. AICD, originally described in tumour immunity, represents a distinct form of cell death driven by ammonia‐mediated disruption of intracellular homeostasis, particularly through coordinated lysosomal and mitochondrial damage [12]. Despite its recognised cytotoxicity, the relevance of AICD to degenerative diseases such as IVDD remains unexplored. Here, we extend the concept of AICD to disc degeneration and provide evidence that ammonia stress induces a non‐canonical cell death phenotype in NPCs that is mechanistically distinct from classical PCD pathways. Functionally, our findings demonstrate that inhibition of apoptosis, ferroptosis, pyroptosis, or necroptosis fails to fully rescue ammonia‐induced NP cell death, supporting its distinction from these canonical modalities. Notably, pharmacological inhibition across different PCD pathways confers variable degrees of protection, indicating that AICD cannot be attributed to any single canonical mechanism. Instead, these differential rescue effects suggest that AICD engages components of multiple cell death programs to varying extents, thereby reflecting a spectrum of mechanistic interconnections rather than a discrete pathway. Importantly, temporal analyses further reveal that ammonia‐induced cellular perturbations precede the activation of key markers associated with canonical PCD, supporting a model in which AICD represents an early, upstream response that may subsequently intersect with or potentiate other death pathways. Together with subcellular evidence of coordinated lysosomal–mitochondrial disruption, these findings provide multi‐dimensional support (functional, temporal, and organelle‐level) for AICD as a primary response to ammonia stress. Moreover, these findings can be better understood in light of the distinct execution mechanisms underlying canonical programmed cell‐death pathways. Apoptosis is primarily mediated by caspase activation, ferroptosis by iron‐dependent lipid peroxidation, pyroptosis by gasdermin‐mediated membrane pore formation, and necroptosis by RIPK1–RIPK3–MLKL signalling. Despite these mechanistic distinctions, the variable and incomplete protection afforded by pathway‐specific inhibitors suggests that these programs may be concurrently engaged rather than acting in isolation during ammonia‐induced NP cell death. Collectively, our findings support the concept of AICD as a previously unrecognised, metabolically driven, and potentially upstream cell‐death process in IVDD, expanding the current understanding of its relationship with canonical PCD pathways and highlighting an important role for metabolic stress in coordinating multiple cell‐death programs in NPCs.

Disruption of organelle homeostasis and inter‐organelle communication is increasingly recognised as a fundamental mechanism underlying cell death beyond canonical signalling cascades [53]. On one hand, dysfunction of individual organelles can directly trigger distinct forms of cell death. On the other hand, the coordination among organelles constitutes a higher‐order regulatory network that integrates metabolic flux, stress responses, and quality control. Together, these two dimensions define a critical layer of cell fate regulation that is increasingly implicated in degenerative diseases [54, 55]. Representative studies further support a causal role for organelle dysfunction in driving cell death programs. Lysosomal destabilisation, for instance, has long been recognised as a driver of lysosome‐dependent cell death, as exemplified by ATP13A2 deficiency–mediated disruption of lysosomal polyamine export and integrity [56]. In contrast, mitochondrial dysfunction has more recently been implicated in newly defined lytic cell death modalities such as mitoxyperiosis, in which metabolic and innate immune signals induce mitochondria‐dependent membrane rupture [57]. These findings collectively underscore that organelle damage is not merely secondary to stress but can serve as a primary driver of cell death. Beyond individual organelles, emerging evidence further emphasises the importance of inter‐organelle crosstalk in maintaining cellular homeostasis. Dynamic communication among mitochondria, lysosomes, endoplasmic reticulum, and other organelles is essential for establishing compartmentalised metabolic homeostasis and coordinating adaptive stress responses [58, 59, 60]. Disruption of this interconnected network has emerged as a hallmark of ageing‐associated degenerative disorders, underscoring the vulnerability of organelle communication systems to metabolic perturbations [61]. Within this framework, our study reveals that ammonia stress induces a coordinated breakdown of organelle homeostasis in NPCs. Multi‐omics analyses first revealed coordinated alterations in pathways governing lysosomal function, mitochondrial metabolism, oxidative stress, and autophagy, which were subsequently validated in vitro and in vivo. Mechanistically, ammonia exposure leads to lysosomal impairment, reflected by disrupted acidic compartments and reduced degradative capacity. Concurrently, mitochondrial dysfunction is characterised not only by excessive reactive oxygen species accumulation but also by a metabolic shift from oxidative phosphorylation towards glycolysis, indicating a disruption of bioenergetic homeostasis that further amplifies intracellular stress. Critically, ammonia also suppresses mitophagic flux, resulting in defective mitochondrial quality control and the accumulation of damaged mitochondria. Together, these events form a self‐reinforcing cycle in which lysosomal alkalinisation compromises lysosomal degradation and autophagosome–lysosome fusion, mitochondrial dysfunction exacerbates oxidative stress, and impaired PINK1–Parkin‐dependent mitophagic flux limits the efficient clearance of damaged mitochondria. The reciprocal amplification of these abnormalities further destabilises lysosomal–mitochondrial homeostasis and ultimately promotes the activation of downstream cell‐death pathways.

Despite the comprehensive findings presented, several limitations should be acknowledged. To begin with, the classification of IVDD severity was primarily based on imaging criteria, and obtaining well‐matched human samples in terms of age, sex, and body mass index remained challenging. This heterogeneity may introduce potential confounding factors, and thus the findings should be interpreted with caution. Secondly, although the rat model of ammonia accumulation recapitulated key pathological features of IVDD under ammonia stress, metabolic differences between rodents and humans may limit its translational applicability, warranting further validation in large animal models with metabolic systems more comparable to humans.

Additionally, although circulating ammonia is clinically accessible and associated with IVDD severity, it may not accurately reflect local ammonia accumulation within the disc. Direct measurement of intradiscal ammonia and its association with IVDD would provide more compelling evidence in future studies. Finally, while this study elucidates AICD from the perspective of organelle crosstalk, the upstream regulatory targets remain insufficiently defined. Ongoing studies are investigating these targets and will be reported in future work.

In summary, this study delineates a previously unrecognised mechanism by which ammonia accumulation drives NP cell death through coordinated disruption of organelle homeostasis. By establishing a functional link between ammonia stress and organelle crosstalk, our findings define AICD as a distinct pathological paradigm in IVDD. This work not only advances the current understanding of metabolic stress–mediated disc degeneration but also highlights the organelle network as a critical regulatory hub in this process. Importantly, these findings suggest that targeting ammonia metabolism or restoring organelle homeostasis may represent promising therapeutic strategies for IVDD, providing a conceptual framework for future metabolism‐based interventions.

## Author Contributions

**Yang Zhang:** conceptualization, methodology, validation, writing – original draft. **Yucheng Gao:** data curation, formal analysis, visualization. **Shuhang Dong:** formal analysis, visualization. **Shuanggong Liu:** data curation. **Lei Liu:** visualization. **Ziyi Song:** data curation. **Jie Xu:** validation. **Yijun Rong:** formal analysis. **Guangxu Song:** visualization. **Yingze Zhang:** resources, project administration. **Dongjin Wu:** resources, project administration. **Zengxin Gao:** conceptualization, writing – review and editing, resources, project administration.

## Funding

This work was supported by National Key Research and Development Program of China (2025YFC2511603) and Natural Science Foundation of Shandong Province (ZR2025MS1525).

## Ethics Statement

This study was approved by the Ethics Committee of the Medical School of Southeast University (ethics approval number: 2025ZDKYSB039). All patients signed informed consent forms. The animal experiments were conducted with prior review and approval from the Institutional Animal Care and Use Committee (IACUC) of the Second Qilu Hospital of Shandong University (ethics approval number: KYLL2025694).

## Conflicts of Interest

The authors declare no conflicts of interest.

## Supporting information


**Figure S1:** (A) Quantitative analysis of immunohistochemical staining for Col II, GLS1, and GLUL. (B) Representative H&E and Safranin O/Fast Green staining of rat intervertebral discs at 2 and 12 months of age. (C) Quantitative analysis of immunofluorescence staining for GLUD1 and GLUL in rat intervertebral disc tissues. (D) Quantitative analysis of immunofluorescence staining for GLS1 and GLUL in HNPCs under different treatments.
**Figure S2:** (A) Orthogonal partial least squares discriminant analysis (OPLS‐DA) score plot of metabolomic profiles between groups. (B) Permutation test of the OPLS‐DA model. (C) Classification of differential metabolites based on chemical properties.
**Figure S3:** (A) Measurement of serum ammonia levels in rats from different groups. (B) Representative H&E staining of major organs from rats in different groups. Data are presented as mean ± SD. Statistical significance is indicated as follows: *p < 0.05, **p < 0.01, ***p < 0.001, ****p < 0.0001; ns, not significant.
**Figure S4:** (A) Quantitative analysis of EdU staining in NPCs. (B) Quantitative analysis of Calcein‐AM/PI staining in NPCs.
**Figure S5:** (A) Quantitative analysis of TUNEL staining in NPCs. (B) Quantitative analysis of apoptosis assessed by flow cytometry in NPCs. (C) Quantitative analysis of immunofluorescence co‐staining for Bax and cleaved caspase‐3 in NPCs.
**Figure S6:** (A) Protein levels of ADAMTS‐4 and ADAMTS‐5 in nucleus pulposus cells across experimental groups, assessed by Western blot. (B) Densitometric quantification of Western blot signals. Data are presented as mean ± SD. Statistical significance is indicated as follows: *p < 0.05, **p < 0.01, ***p < 0.001, ****p < 0.0001; ns, not significant.
**Figure S7:** (A) Relative quantification of IL‐1β and IL‐6 co‐immunofluorescence in nucleus pulposus cells. (B) Relative quantification of β‐galactosidase staining. (C) Relative quantification of ACAN and ADAMTS‐5 co‐immunofluorescence.
**Figure S8:** (A) Quantification of mitochondrial staining using Mito‐Tracker. (B) Quantitative analysis of JC‐1 staining for mitochondrial membrane potential.
**Figure S9:** (A) Relative levels of mtDNA in nucleus pulposus cells across experimental groups, assessed by qPCR. (B) Relative ATP levels in nucleus pulposus cells measured using an ATP detection kit.
**Figure S10:** (A) Mito‐SOX staining of mitochondrial superoxide levels in nucleus pulposus cells across experimental groups. (B) Relative quantification of Mito‐SOX fluorescence intensity. Data are presented as mean ± SD. Statistical significance is indicated as follows: *p < 0.05, **p < 0.01, ***p < 0.001, ****p < 0.0001; ns, not significant.
**Figure S11:** (A) ROS levels in nucleus pulposus cells across experimental groups, assessed by ROS staining. (B) Relative quantification of ROS fluorescence intensity. Data are presented as mean ± SD. Statistical significance is indicated as follows: *p < 0.05, **p < 0.01, ***p < 0.001, ****p < 0.0001; ns, not significant.
**Figure S12:** (A) Western blot analysis of the lysosomal proteins LAMP1 and CTSB and the mitochondrial proteins TOMM20 and ATP5A in NPCs exposed to NH_4_Cl for 0, 0.5, 1, 1.5, 2, and 3 h. (B) Densitometric quantification of LAMP1, CTSB, TOMM20, and ATP5A expression normalized to β‐actin. Data are presented as mean ± SD. Statistical significance is indicated as follows: *p < 0.05, **p < 0.01, ***p < 0.001, ****p < 0.0001; ns, not significant.
**Figure S13:** (A) Relative quantification of GPX4, FTH1, LPCAT3, and SLC7A11 protein levels by Western blot. (B) Relative quantification of NLRP3, ASC, Caspase‐1, and GSDMD‐N protein expression by Western blot. (C) Relative quantification of phosphorylated RIPK1, RIPK3, and MLKL by Western blot. (D) Relative quantification of GPX4, FTH1, LPCAT3, and ACSL4 protein levels by Western blot.
**Figure S14:** CB839 partially reverses NH_4_Cl‐induced alterations in ammonia‐metabolizing enzymes in NPCs. (A) Western blot analysis of GLUL and GLUD1 expression in NPCs treated with NH_4_Cl in the presence or absence of CB839 (*n* = 3). (B) Densitometric quantification of GLUL and GLUD1 expression normalized to β‐actin. Data are presented as mean ± SD. Statistical significance is indicated as follows: *p < 0.05, **p < 0.01, ***p < 0.001, ****p < 0.0001; ns, not significant.
**Figure S15:** (A) Relative quantification of NLRP3, ASC, Caspase‐1, and GSDMD‐N protein levels by Western blot. (B) Relative quantification of phosphorylated RIPK1, RIPK3, and MLKL by Western blot. (C) Relative quantification of Bax, Bcl‐2, cleaved Caspase‐3, and cleaved Caspase‐7 by Western blot. (D) Relative quantification of Calcein‐AM/PI staining.
**Figure S16:** (A) Assessment of cell proliferation in nucleus pulposus cells across experimental groups by EdU staining. (B) Relative quantification of EdU‐positive cells.
**Figure S17:** (A) Relative quantification of Col‐II, SOX‐9, MMP‐3, and MMP‐13 protein levels by Western blot. (B) Relative quantification of LAMP‐1, Galectin‐3, TOMM20, and ATP5A by Western blot. (C) Relative quantification of PINK1, Parkin, LC3, and p62 by Western blot.
**Figure S18:** (A) Relative quantification of Galectin‐3, CTSB, p‐DRP1, Parkin, Cle‐caspase3, ACSL4, GSDMD‐N, and p‐MLKL protein levels by Western blot. (B) Quantitative analysis of Mito‐Tracker and Lyso‐Tracker co‐staining in nucleus pulposus cells.
**Figure S19:** Histological evaluation of major organs (heart, lung, liver, spleen, and kidney) from different groups of rats by H&E staining to assess systemic toxicity.
**Figure S20:** (A) Relative quantification of immunofluorescence staining for LAMP‐2 and CTSB in intervertebral disc tissues from rats across experimental groups. (B) Relative quantification of immunofluorescence staining for TOMM20 and COX IV in intervertebral disc tissues from different groups.
**Table S1:** Primer sequences for qRT‐PCR analysis.
**Table S2:** Antibodies of interest in the study.
**Table S3:** Intervertebral disc degeneration assessment scoring system.

## Data Availability

The data that support the findings of this study are available from the corresponding author upon reasonable request.
