# Supplementary material for: Ammonia Accumulation Drives Intervertebral Disc Degeneration by Triggering Ammonia‐Induced Cell Death Through Lysosome–Mitochondria Crosstalk
Source: Cell Prolif. 2026 Sep 27:e70283. Online ahead of print. doi: 10.1111/cpr.70283 (PMC13616829; doi:10.1111/cpr.70283)
Supplement: Supplementary file 1 [file CPR-9999-e70283-s001.docx]

**Full Length Article**

**Ammonia accumulation drives intervertebral disc degeneration by triggering ammonia-induced cell death through lysosome–mitochondria crosstalk**

*Yang Zhang^1#^*, *Yucheng Gao^1#^**, Shuhang Dong^3^, Shuanggong Liu^2^,* *Lei Liu^1^, Ziyi Song^1^, Jie Xu^1^, Yijun Rong^1^, Guangxu Song^2^, Yingze Zhang^3,4^*, Dongjin Wu^2^*, Zengxin Gao^1^**

*^1^**Department of* *Spinal Surgery, Zhongda Hospital, College of Medicine, Southeast University*, *Dingjiaqiao 87,* *Nanjing, Jiangsu210009, People’s Republic of China* *(First Affiliated Institution)*

*^2^Department of Orthopaedics,* *The Second Qilu Hospital of Shandong University, 247 Beiyuan Street, Jinan, Shandong250033, China*

*^3^Department of Orthopedics, The Affiliated Hospital of Qingdao University, Qingdao, Shandong 266000, China*

*^4^Department of Orthopedics, The Third Affiliated Hospital of Hebei Medical University, Shijiazhuang, Hebei 050051, China*

**Correspondence:* *zhangyingzeqdu@163.com; doctorwdj@163.com; zhongdaspinegzx@163.com*

*^#^These authors contributed equally to this work*

**Figure S1.** (A) Quantitative analysis of immunohistochemical staining for Col II, GLS1, and GLUL. (B) Representative H&E and Safranin O/Fast Green staining of rat intervertebral discs at 2 and 12 months of age. (C) Quantitative analysis of immunofluorescence staining for GLUD1 and GLUL in rat intervertebral disc tissues. (D) Quantitative analysis of immunofluorescence staining for GLS1 and GLUL in HNPCs under different treatments.

**Figure S2.** (A) Orthogonal partial least squares discriminant analysis (OPLS-DA) score plot of metabolomic profiles between groups. (B) Permutation test of the OPLS-DA model. (C) Classification of differential metabolites based on chemical properties.

**Figure S3.** (A) Measurement of serum ammonia levels in rats from different groups. (B) Representative H&E staining of major organs from rats in different groups. Data are presented as mean ± SD. Statistical significance is indicated as follows: *p < 0.05, **p < 0.01, ***p < 0.001, ****p < 0.0001; ns, not significant.

**Figure S4.** (A) Quantitative analysis of EdU staining in NPCs. (B) Quantitative analysis of Calcein-AM/ PI staining in NPCs.

**Figure S5.** (A) Quantitative analysis of TUNEL staining in NPCs. (B) Quantitative analysis of apoptosis assessed by flow cytometry in NPCs. (C) Quantitative analysis of immunofluorescence co-staining for Bax and cleaved caspase-3 in NPCs.

**Figure S6.** (A) Protein levels of ADAMTS-4 and ADAMTS-5 in nucleus pulposus cells across experimental groups, assessed by Western blot. (B) Densitometric quantification of Western blot signals. Data are presented as mean ± SD. Statistical significance is indicated as follows: *p < 0.05, **p < 0.01, ***p < 0.001, ****p < 0.0001; ns, not significant.

**Figure S7.** (A) Relative quantification of IL-1β and IL-6 co-immunofluorescence in nucleus pulposus cells. (B) Relative quantification of β-galactosidase staining. (C) Relative quantification of ACAN and ADAMTS-5 co-immunofluorescence.

**Figure S8.** (A) Quantification of mitochondrial staining using Mito-Tracker. (B) Quantitative analysis of JC-1 staining for mitochondrial membrane potential.

**Figure S9.** (A) Relative levels of mtDNA in nucleus pulposus cells across experimental groups, assessed by qPCR. (B) Relative ATP levels in nucleus pulposus cells measured using an ATP detection kit.

**Figure S10.** (A) Mito-SOX staining of mitochondrial superoxide levels in nucleus pulposus cells across experimental groups. (B) Relative quantification of Mito-SOX fluorescence intensity. Data are presented as mean ± SD. Statistical significance is indicated as follows: *p < 0.05, **p < 0.01, ***p < 0.001, ****p < 0.0001; ns, not significant.

**Figure S11.** (A) ROS levels in nucleus pulposus cells across experimental groups, assessed by ROS staining. (B) Relative quantification of ROS fluorescence intensity. Data are presented as mean ± SD. Statistical significance is indicated as follows: *p < 0.05, **p < 0.01, ***p < 0.001, ****p < 0.0001; ns, not significant.

**Figure S12.** (A) Western blot analysis of the lysosomal proteins LAMP1 and CTSB and the mitochondrial proteins TOMM20 and ATP5A in NPCs exposed to NH₄Cl for 0, 0.5, 1, 1.5, 2, and 3 h. (B) Densitometric quantification of LAMP1, CTSB, TOMM20, and ATP5A expression normalized to β-actin. Data are presented as mean ± SD. Statistical significance is indicated as follows: *p < 0.05, **p < 0.01, ***p < 0.001, ****p < 0.0001; ns, not significant.

**Figure S13.** (A) Relative quantification of GPX4, FTH1, LPCAT3, and SLC7A11 protein levels by Western blot. (B) Relative quantification of NLRP3, ASC, Caspase-1, and GSDMD-N protein expression by Western blot. (C) Relative quantification of phosphorylated RIPK1, RIPK3, and MLKL by Western blot. (D) Relative quantification of GPX4, FTH1, LPCAT3, and ACSL4 protein levels by Western blot.

**Figure S14. CB839 partially reverses NH₄Cl-induced alterations in ammonia-metabolizing enzymes in NPCs.** (A) Western blot analysis of GLUL and GLUD1 expression in NPCs treated with NH₄Cl in the presence or absence of CB839 (n=3). (B) Densitometric quantification of GLUL and GLUD1 expression normalized to β-actin. Data are presented as mean ± SD. Statistical significance is indicated as follows: *p < 0.05, **p < 0.01, ***p < 0.001, ****p < 0.0001; ns, not significant.

**Figure S15**. (A) Relative quantification of NLRP3, ASC, Caspase-1, and GSDMD-N protein levels by Western blot. (B) Relative quantification of phosphorylated RIPK1, RIPK3, and MLKL by Western blot. (C) Relative quantification of Bax, Bcl-2, cleaved Caspase-3, and cleaved Caspase-7 by Western blot. (D) Relative quantification of Calcein-AM/PI staining.

**Figure S16.** (A) Assessment of cell proliferation in nucleus pulposus cells across experimental groups by EdU staining. (B) Relative quantification of EdU-positive cells.

**Figure S17.** (A) Relative quantification of Col-II, SOX-9, MMP-3, and MMP-13 protein levels by Western blot. (B) Relative quantification of LAMP-1, Galectin-3, TOMM20, and ATP5A by Western blot. (C) Relative quantification of PINK1, Parkin, LC3, and p62 by Western blot.

**Figure S18.** (A) Relative quantification of Galectin-3, CTSB, p-DRP1, Parkin, Cle-caspase3, ACSL4, GSDMD-N, and p-MLKL protein levels by Western blot. (B) Quantitative analysis of Mito-Tracker and Lyso-Tracker co-staining in nucleus pulposus cells.

**Figure S19.** Histological evaluation of major organs (heart, lung, liver, spleen, and kidney) from different groups of rats by H&E staining to assess systemic toxicity.

**Figure S20.** (A) Relative quantification of immunofluorescence staining for LAMP-2 and CTSB in intervertebral disc tissues from rats across experimental groups. (B) Relative quantification of immunofluorescence staining for TOMM20 and COX IV in intervertebral disc tissues from different groups.

**Table S1**. Primer sequences for qRT-PCR analysis

| ***Gene*** | ***Forward primer*** | ***Reverse primer*** |
| --- | --- | --- |
| **Col-II** | 5'- GGCAATAGCAGGTTCACGTACA-3’ | 5'- CGATAACAGTCTTGCCCCACTT-3’ |
| **SOX-9** | 5'- ACGCCGAGCTCAGCAAGA-3’ | 5'- CACGAAGGGCCGCTTCT-3’ |
| **MMP-3** | 5'- GATGCCCACTTTGATGATGAA-3’ | 5'- AGTGTTGGCTGAGTGAAAGAGACC-3’ |
| **GLUD1** | 5'- GGGCTTTTGTATCCGTTACAGC-3’ | 5'- GGTCAAACGGCCATAGCTGA-3’ |
| **GLS1** | 5'- TCAGCGTGTCCAAACACTGAG-3’ | 5'- CGCCAAGGGAGTTAAAGACTT-3’ |
| **GLUL** | 5'- TCATCTTGCATCGTGTGTGTG-3’ | 5'- CTTCAGACCATTCTCTCCGG-3’ |
| **mtDNA** | 5'- GCACCTACCCTATCACTC-3’ | 5'- TTGTTTGGGCTACGGCTC-3’ |
| **β-actin** | 5'- TGTCCACCTTCCAGCAGATGT-3’ | 5'- AGCTCAGTAACAGTCCGCCTAG-3’ |

**Table S2**. Antibodies of interest in the study

| ***Antibodies*** | ***Source*** | ***Species*** | ***Application*** | ***Dilution rate*** |
| --- | --- | --- | --- | --- |
| **GLUD1** | Affinity | Rabbit | WB | 1:1000 |
|  |  | Rabbit | IF/IHC | 1:200 |
| **GLS1** | Abcam | Rabbit | WB | 1:1000 |
|  |  | Rabbit | IF/IHC | 1:250 |
| **GLUL** | Cell Signaling Technology | Rabbit | WB | 1:1000 |
|  |  | Rabbit | IF/IHC | 1:100 |
| **Aggrecan** | Cell Signaling Technology | Rabbit | IF/IHC | 1:200 |
| **Col-II** | Affinity | Rabbit | WB | 1:2000 |
|  |  | Rabbit | WB | 1:250 |
| **SOX-9** | Affinity | Rabbit | WB | 1:1000 |
| **MMP-3** | Affinity | Rabbit | WB | 1:1000 |
|  |  | Rabbit | IHC | 1:250 |
| **MMP-13** | Affinity | Rabbit | WB | 1:1000 |
|  |  | Rabbit | IHC | 1:250 |
| **ADAMTS-4** | Abcam | Rabbit | WB | 1:2000 |
| **ADAMTS-5** | Abcam | Rabbit | WB | 1:1000 |
|  |  | Rabbit | IF | 1:200 |
| **IL-6** | Cell Signaling Technology | Rabbit | IF | 1:200 |
| **IL-β** | Cell Signaling Technology | Rabbit | IF | 1:300 |
| **Bax** | Affinity | Rabbit | WB | 1:1000 |
|  |  | Rabbit | IF/IHC | 1:250 |
| **Bcl-2** | Cell Signaling Technology | Mouse | WB | 1:1000 |
| **Cle-caspase3** | Cell Signaling Technology | Rabbit | WB | 1:1000 |
|  |  |  | IF/IHC | 1:400 |
| **Cle-caspase7** | Cell Signaling Technology | Rabbit | WB | 1:1000 |
| **LAMP-1** | Cell Signaling Technology | Rabbit | WB | 1:1000 |
|  |  | Rabbit | IF/IHC | 1:200 |
| **LAMP-2** | Cell Signaling Technology | Rabbit | WB | 1:1000 |
| **TRPML1** | Abcam | Rabbit | WB | 1:1000 |
| **Galectin-3** | Affinity | Rabbit | WB | 1:1000 |
| **Cathepsin-B** | Abcam | Rabbit | WB | 1:1000 |
|  |  | Rabbit | IF/IHC | 1:250 |
| **V-ATPase** | Affinity | Rabbit | WB | 1:1000 |
| **TOMM20** | Cell Signaling Technology | Rabbit | WB | 1:1000 |
|  |  | Rabbit | IF/IHC | 1:200 |
| **VDAC1** | Affinity | Rabbit | WB | 1:1000 |
| **S-OPA1** | Affinity | Rabbit | WB | 1:1000 |
| **ATP5A** | Affinity | Mouse | WB | 1:1000 |
| **Cyt-C** | Affinity | Rabbit | WB | 1:1000 |
| **p-DRP1** | Affinity | Rabbit | WB | 1:1000 |
| **COX IV** | Abcam | Rabbit | IHC | 1:200 |
| **SLC7A11** | Abcam | Rabbit | WB | 1:1000 |
|  |  | Rabbit | IHC | 1:200 |
| **PINK1** | Affinity | Rabbit | WB | 1:1000 |
|  |  | Rabbit | IHC | 1:100 |
| **Parkin** | Affinity | Rabbit | WB | 1:1000 |
|  |  | Rabbit | IF | 1:200 |
| **LC3B** | Cell Signaling Technology | Mouse | WB | 1:1000 |
|  |  | Mouse | IF/IHC | 1:200 |
| **P62** | Cell Signaling Technology | Rabbit | WB | 1:1000 |
| **Atg5** | Affinity | Rabbit | WB | 1:1000 |
| **Atg12** | Cell Signaling Technology | Rabbit | WB | 1:1000 |
| **GPX4** | Affinity | Rabbit | WB | 1:1000 |
| **FTH1** | Affinity | Rabbit | WB | 1:1000 |
| **LPCAT3** | Cell Signaling Technology | Rabbit | WB | 1:1000 |
| **ACSL4** | Affinity | Rabbit | WB | 1:1000 |
| **NLRP3** | Affinity | Rabbit | WB | 1:1000 |
| **ASC** | Cell Signaling Technology | Rabbit | WB | 1:1000 |
| **Caspase-1** | Affinity | Rabbit | WB | 1:1000 |
| **GSDMD-N** | Affinity | Rabbit | WB | 1:1000 |
| **p-RIPK1** | Affinity | Rabbit | WB | 1:1000 |
| **RIPK1** | Affinity | Rabbit | WB | 1:1000 |
| **p-RIPK3** | Affinity | Rabbit | WB | 1:1000 |
| **RIPK3** | Affinity | Rabbit | WB | 1:1000 |
| **p-MLKL** | Affinity | Rabbit | WB | 1:1000 |
| **MLKL** | Affinity | Rabbit | WB | 1:1000 |
| **Rabbit Secondary antibody** | Cell Signaling Technology | Goat | WB | 1:10000 |
| **Mouse Secondary antibody** | Cell Signaling Technology | Goat | WB | 1:10000 |
| **β-actin** | Cell Signaling Technology | Rabbit | WB | 1:1000 |

**Table S3**. Intervertebral disc degeneration assessment scoring system

| ***Score*** | ***Nucleus pulposus*** | ***Annulus fibrosus*** | ***Osteophyte*** |
| --- | --- | --- | --- |
| **0** | Bulging gel with abundant | Compact fibrous lamellas | Absence |
|  | notochordal cells |  |  |
| **1** | Notochordal cells loss; | Proliferation of fibrocartilaginous tissue | Appearance |
|  | chondrocyte-like cells emergence | and loss of nuclear-annular border |  |
| **2** | Focal mucoid degeneration; clefts | Fissures in annulus fibrosis | Overgrowth |
| **3** | Diffuse mucoid degeneration and |  |  |
|  | clefts throughout nucleus |  |  |
